# A Risk Analysis of the Release of Liquid Hydrogen in Road Tunnels: The Effects of Mechanical Ventilation Combined With Geometric and Traffic Characteristics

**DOI:** 10.1111/risa.70157

**Published:** 2025-11-20

**Authors:** Ciro Caliendo, Gianluca Genovese, Isidoro Russo

**Affiliations:** ^1^ Department of Civil Engineering University of Salerno Fisciano Salerno Italy

**Keywords:** computational fluid dynamics, liquid hydrogen transport, probit function, risk analysis, road tunnels

## Abstract

The transportation of liquid hydrogen (LH_2_) via road tankers could prove to be the most cost‐effective short‐term option for long‐distance delivery. However, there are significant risks, particularly in confined spaces like road tunnels. An accidental release of LH_2_ in these structures is likely to create a flammable hydrogen cloud, the explosion of which generates overpressures whose magnitude depends on several mutually dependent variables, including geometry, traffic, and ventilation. Nevertheless, the combined effect of the above‐mentioned variables on user safety in the event of an accidental leakage and explosion of LH_2_ from a road tanker in a tunnel has yet to be investigated in detail. This study develops 3D CFD models of both the release and explosion of LH_2_ to address this issue, along with a comprehensive parametric analysis that considers different tunnel lengths, negative and positive longitudinal slopes, traffic volumes, and ventilation types (i.e., natural or longitudinal mechanical). The CFD code used was preliminarily calibrated against experimental literature tests. Subsequently, a risk analysis was carried out using the CFD results in terms of overpressures, which, combined with a probit function, made it possible to estimate the number of potential fatalities. Consequently, a probability matrix of the risk of having a given number (*N*) of fatalities was built as a function of the tunnel length, ventilation type (i.e., natural or mechanical), longitudinal slope, and traffic volume. The results revealed the benefits of positive gradients as well as of implementing a longitudinal mechanical ventilation system. In contrast, longer tunnels increase the probability of having a given number of fatalities. This study might serve as a reference for tunnel operators in the choice of mitigation measures and/or traffic control strategies to limit the negative consequences of the release of liquid hydrogen in road tunnels.

## 1 Introduction

Among the key objectives of the European Green Deal (2019) on climate change there are: (i) the reduction of greenhouse gas emissions by 50%–55% by 2030 compared to 1990 levels; (ii) climate neutrality by 2050; (iii) the increased use of clean and renewable energy; (iv) the improvement of energy efficient in buildings, transport, and industries; (iv) the promotion of sustainable mobility. In this context, hydrogen is a promising new energy carrier (NEC) in the transition to a low‐carbon economy since it can be produced by renewable sources and does not emit any greenhouse gases when used, thus contributing to climate change mitigation.

In 2021, world hydrogen consumption was about 94 million tons with an increase of around 5% compared to 2020. It is expected to grow up to about 130 million tons by 2030 to achieve longer‐term net zero goals (Halder et al. 2024). Nevertheless, as the use of hydrogen becomes more widespread, safety concerns about its transport from production sites to consumers are increasing due to its well‐known hazardous nature (e.g., low ignition energy, high flammability range, and high heat of combustion) (Karplus et al. 2024). In this respect, hydrogen might be transported in different ways, such as via road, rail, pipelines, and water. Despite a pipeline delivery system having the lowest pollution emissions among the other modes of distribution, a high‐pressure hydrogen pipeline network requires an accurate selection of the materials used as well as substantial investments, which are not feasible in the short‐term perspective (Mahajan et al. 2022). Therefore, alternative and more cost‐effective means of transport such as road trucks are now used to deliver hydrogen, especially in liquid form. When hydrogen is liquified, it occupies much less space than its gaseous form, thus making its transport more convenient (i.e., much larger quantities can be carried), particularly over long distances (Ball and Wietschel 2009; Hall et al. 2014). However, the transport of liquid hydrogen (LH_2_) by road has several risks, especially those associated with leaks and explosions that could threaten human life (Han et al. 2024). Accidental releases of LH_2_ are particularly challenging in confined environments such as road tunnels where it is likely to accumulate under the ceiling while potentially creating a flammable mixture with air that might lead to catastrophic explosions (Utgikar and Thiesen 2005; Kožuh 2014). Therefore, the accurate assessment of the consequences for users of accidental releases and explosions of LH_2_ in road tunnels is now a topic of significant interest to the scientific community and competent authorities at different levels. This paper falls into this context.

The magnitude of the impact on user safety caused by accidental releases and possible explosions of LH_2_ in road tunnels is expected to change as a function of several mutually dependent variables, such as: (i) the tunnel length: in longer tunnels, especially when the accidental event occurs far from the portals, the flammable hydrogen–air mixture (i.e., the hydrogen cloud) might have, for example, more time to expand before the arrival of rescue teams; (ii) the vertical alignment of the tunnel: the movement of the hydrogen cloud is affected by the longitudinal slope, tending to follow the downward direction in the case of positive gradients (i.e., the chimney effect) or the upward direction in the presence of negative inclinations (i.e., the inverse chimney effect); (iii) the traffic volume passing through the tunnel: the vehicles in the queue act as obstacles to the dispersion of the released hydrogen, promoting its accumulation inside the structure; (iv) the ventilation conditions along the tunnel: the presence of a mechanical ventilation system might play a key role by facilitating the dilution of the hydrogen cloud. Nevertheless, the combined effect of the above‐mentioned factors on the safety of the tunnel occupants has yet to be investigated in detail, thus representing a gap in our knowledge that this paper will attempt to fill.

In light of the above considerations, the main aim of this study is to examine the combined effect of the geometric, traffic, and ventilation characteristics of a road tunnel on user safety in the event of accidental releases and possible explosions of LH_2_ from a road tanker. 3D computational fluid dynamics (CFD) models of LH_2_ dispersion and potential explosions were developed using the ANSYS Fluent code (ANSYS Fluent User Guide 2023). A comprehensive parametric analysis was also carried out. The simulations were performed by varying the length and longitudinal slope of a two‐lane unidirectional road tunnel assumed to be under different ventilation conditions (i.e., natural or longitudinal mechanical), while also considering different traffic volumes. Subsequently, a risk analysis was carried out using the CFD results in terms of overpressures, which, combined with a probit function, made it possible to compute the number of potential victims, as well as to develop a probability matrix of the risk of having a given number of fatalities.

The paper is structured as follows: Section 2 includes a short review of some experimental and/or numerical studies on hydrogen releases and explosions in confined spaces. In Section 3, the methodology and parametric analysis are introduced. Subsequently, the CFD models are presented in Section 4, and their results are reported and commented on in Section 5. Finally, some conclusions and considerations for practical applications are drawn in Section 6, along with future research directions.

## 2 Literature Review

In the last two decades, several studies involving both experimental tests and numerical simulations have been carried out on hydrogen releases and explosions in confined spaces, including road tunnels, primarily with the aim of understanding their behavior and identifying potential mitigation measures.

A number of works have focused on the role of ventilation. While some found that ventilation had a limited impact on maximum overpressures during explosions (Middha and Hansen 2009), others reported that increased longitudinal ventilation can influence hydrogen distribution and reduce average overpressures (Bie and Hao 2017). Similarly, mechanical ventilation was shown to enhance hydrogen diffusion and reduce the volume of flammable clouds, thereby mitigating potential risks (Cui et al. 2023; Yan et al. 2024; Hu et al. 2025).

The geometry of the tunnel and presence of obstacles have also been shown to significantly influence explosion dynamics. Increased aspect ratios and the presence of obstacles, such as vehicles, intensify flame propagation and overpressures due to enhanced turbulence and flame‐pressure interactions (Tolias et al. 2014; Molkov and Dery 2020; Qin and Chen 2021; Shen et al. 2021; Kudriakov et al. 2022; Han et al. 2024; Wang et al. 2025).

Other studies investigated the effect of hydrogen inventory and release characteristics. Larger leakages lead to more extensive flammable clouds in both vertical and horizontal directions (Middha and Hansen 2009; Wang et al. 2025).

In general, hydrogen explosions in tunnels—whether involving compressed or LH_2_—can result in severe consequences for tunnel users, depending on ignition source, cloud concentration, and environmental conditions (Li 2019; Tang et al. 2020; Hu et al. 2023). In this context, CFD models have proven to be the most suitable tool for investigating hydrogen dispersion and explosion dynamics, as well as for evaluating mitigation measures in confined environments such as road tunnels. The reliability of CFD models has been supported through various validation efforts. (Kudriakov et al. 2022; Hansen and Hansen 2023; Caliendo et al. 2024).

Table 1 summarizes the methods and objectives of all the aforementioned studies, while also indicating the variables (e.g., hydrogen cloud size, ignition source, ventilation, etc.) eventually analyzed in each of them.

**TABLE 1:** Summary of the literature review.

Reference	Method(s)	Variable(s)	Objective
Middha and Hansen (2009)	Modeling	Hydrogen inventory and ventilation, as well as cross‐section shape	To assess the effect of hydrogen inventory and ventilation on the overpressures in tunnels
Tolias et al. (2014)	Modeling	Presence and absence of vehicles	To investigate the impact of vehicles on explosion hazards in tunnels
Bie and Hao (2017)	Modeling	Ventilation and size of the flammable hydrogen cloud	To evaluate the influence of longitudinal ventilation and flammable hydrogen cloud size on the overpressures in tunnels
Y. Z. Li (2019)	Review of studies	—	To analyze the hazards of alternative fueled vehicles in tunnels, including hydrogen vehicles
Tang et al. (2020)	Modeling	—	To investigate the hazards of accidental releases of liquid hydrogen in confined spaces, including tunnels
Molkov and Dery (2020)	Modeling	Cross‐section areas, tunnel length, and tanks volumes	To develop engineering correlations for the blast wave decay in tunnels after the catastrophic rupture of the hydrogen tank
Shen et al. (2021)	Modeling and experiment	Aspect ratio	To assess the impact of aspect ratio on hydrogen‐air explosions in closed channels
Qin and Chen (2021)	Modeling	Number of obstacles	To examine the effect of obstacles on hydrogen‐air explosions in closed ducts
Kudriakov et al. (2022)	Experiment	Hydrogen gas pressure	To validate engineering correlations on hydrogen blast wave overpressures in tunnels
Hu et al. (2023)	Review of studies	—	To highlight the strengths and weaknesses of CFD tools for hydrogen explosions
Cui et al. (2023)	Modeling	Release directions and sites, as well as ambient wind conditions	To simulate the explosion after an accidental leakage of hydrogen in a real‐size road tunnel
Hansen and Hansen (2023)	Modeling and experiment	Indoor and outdoor environment	To study liquid hydrogen dispersion and explosion properties
Han et al. (2024)	Experiment	Hydrogen volume fractions and obstacles	To evaluate the effect of hydrogen volume fraction and obstacles on flame propagation and overpressures in confined spaces
Yan et al. (2024)	Modeling	Leakage mass flow rate and area, traffic congestion conditions, and ventilation	To examine the diffusion characteristics of hydrogen gas clouds after leakage in tunnel spaces
Caliendo et al. (2024)	Modeling	Ventilation and off or peak traffic hours	To quantify the effects on the user safety caused by both LH_2_ release and potential deflagration and/or detonation inside road tunnels
Hu et al. (2025)	Modeling	Extracted ventilation and ambient humidity	To investigate the diffusion and explosion behavior of LH_2_ leaks in tunnels.
Wang et al. (2025)	Modeling	Leakage volume and blockage ratio	To study the diffusion behavior and explosible risk of LH_2_ leakage in tunnels

However, the above‐mentioned chronological literature review shows that the entirety of benefits due to mechanical ventilation on the tunnel safety in the event of a LH_2_ release and possible consequent explosion—when compared to the natural one, and as a function of geometry and traffic conditions—has not been sufficiently investigated. This gap of knowledge will be now filled by the present paper. By developing a probabilistic risk matrix, our research provides also practical applications for tunnel operators in the choice of more appropriate mitigative measures and/or traffic control strategies to limit the negative consequences of the release of LH_2_ in road tunnels.

## 3 Materials and Methods

### 3.1 Road Tunnel Characterization

#### 3.1.1 Length and Cross‐Section

**FIGURE 1:**
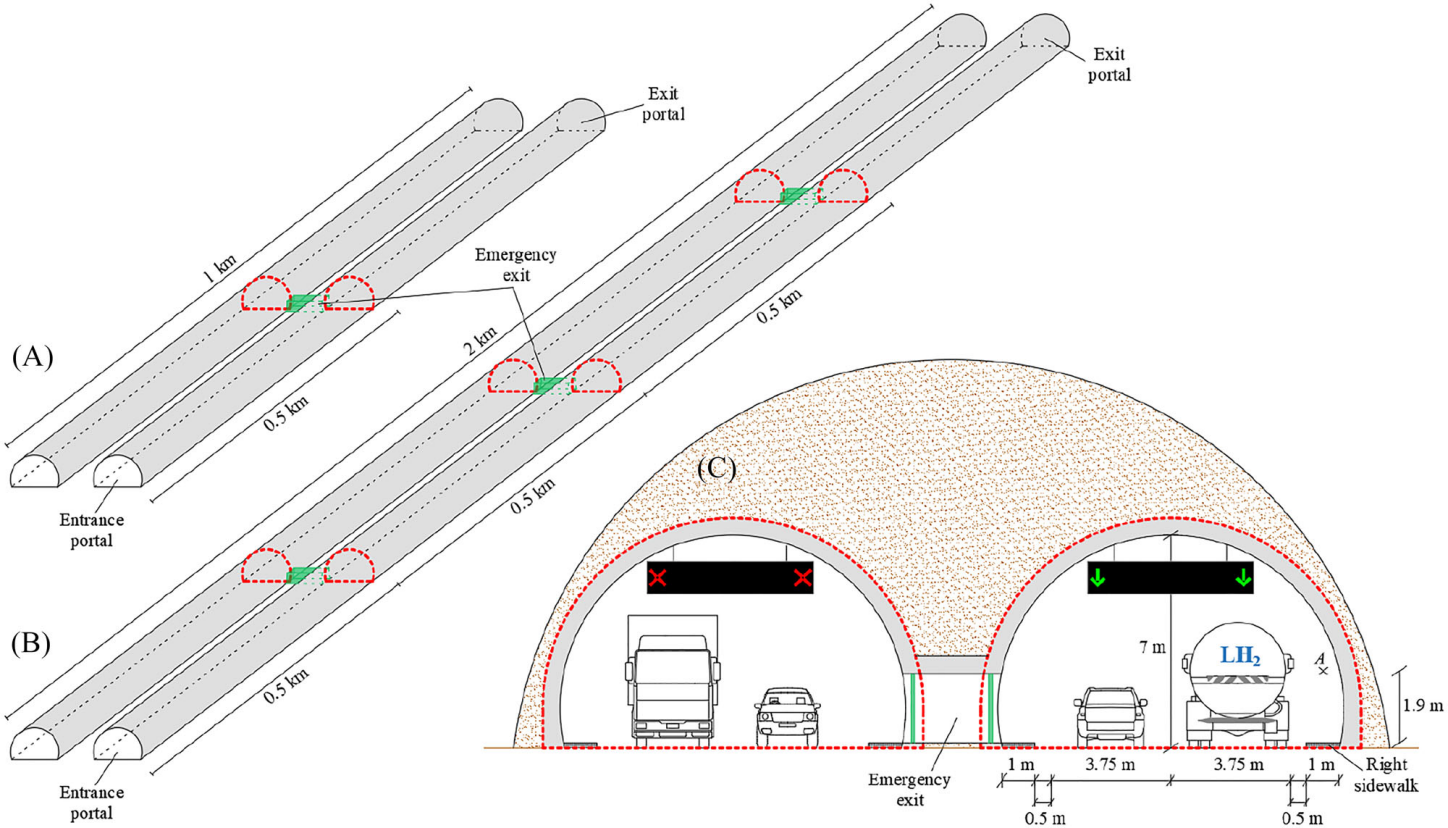
Schematic representation of the (a) 1 km and (b) 2 km long two‐tube road tunnels under investigation, as well as (c) the corresponding cross‐section, in which each tube is assumed to be characterized by unidirectional traffic and the accidental release of LH_2_ occurs in a tube.

An existing two‐tube road tunnel located along the Italian motorway network was taken as a reference for this study. The tunnel tube is straight and has a horseshoe‐shaped cross‐section of 63 m^2^ with a maximum height and width of 7 and 10.5 m, respectively. Figure 1 shows the cross‐section of the investigated two‐tube road tunnel, in which each tube is assumed to be characterized by unidirectional traffic and the accidental release of LH_2_ occurs in a tube. An emergency exit connecting the two tubes is also located in the middle of the tunnel length (i.e., 500 m from the entrance portal of the tunnel). Moreover, to assess the impact of the tunnel length (*L*) on user safety in the event of accidental releases and possible explosions of LH_2_, numerical simulations were also conducted considering a 2 km‐long tunnel having the same above‐mentioned geometric characteristics. In this case, the emergency exits connecting the two tubes are assumed to be located at every 500 m in accordance with the European Commission (2004), which was also adopted by the Italian Ministry of Infrastructure and Transport (2006).

#### 3.1.2 Vertical Alignment

Longitudinal slope plays a crucial role for user safety in road tunnels, primarily influencing the propagation along the structure of toxic gases and temperatures in the event of a fire or the development of the flammable hydrogen cloud during an accidental release of LH_2_, as in this case. Therefore, to investigate the influence of the vertical alignment on the safety of the tunnel occupants in the event of accidental releases and possible explosions of LH_2_, the parametric analysis was conducted by varying the longitudinal slope (*i*) of the tunnel between +4% and −4% (i.e., +4, +2, 0, −2, or −4%); where the maximum longitudinal slope equal to 4% is the limit fixed by the Italian legislation (Italian Ministry of Infrastructures and Transports 2001) to contain the emissions of pollutants in road tunnels due to traffic.

#### 3.1.3 Traffic

To evaluate the impact of traffic on user safety in the case of accidental releases and potential explosions of LH_2_, three different peak hourly volumes (PHV*s*) of the unidirectional traffic flow transiting through the incidental tunnel tube were considered in the analysis: 1100, 1750, and 2400 vehicles/h per lane. It is worth mentioning that 2400 vehicles/h is the capacity per lane established by the Highway Capacity Manual (2010) for roads having similar characteristics to the one under consideration, therefore the congested traffic condition was also investigated. The percentage of heavy goods vehicles (HGVs) within the overall traffic was assumed to be 25% (including 2% of buses).

#### 3.1.4 Ventilation

The type of ventilation (i.e., natural or mechanical) inside the road tunnels depends on their length. According to the European Commission (2004), a mechanical ventilation system must be installed in road tunnels with a length (*L*) > 1 km. This means that when *L* is ≤ 1 km, the tunnel might be naturally or mechanically ventilated depending on the safety level required to be ensured for tunnel users. In this paper, therefore, the investigated 2 km long tunnel was considered to be mechanically ventilated, while the 1 km long tunnel was assumed to be either naturally or mechanically ventilated.

The mechanical ventilation system in each tube is assumed to be longitudinal and consists of eight or five axial jet fans located along the ceiling centerline for the 2 or 1 km long tunnel. Each jet fan has a length of 2 m, with a diameter of 0.7 m and a maximum velocity (capacity) of 30 m/s. The first jet fan is located at 125 m from the entrance portal in each scenario examined, while the others are assumed to be spaced at intervals of 200 or 150 m along the 2 or 1 km long tunnel. The mechanical ventilation system is activated after 60 s from the accidental release of LH_2_ from the road tanker.

In the case of natural ventilation (i.e., *L* ≤ 1 km), a positive pressure difference of +5 Pa is imposed between the entrance and exit portals of the tunnel considering the piston effect due to the vehicles in motion (Caliendo et al. 2021).

#### 3.1.5 Material Properties

Table 2 shows the type and characteristics of the material considered for the tunnel structure and road pavement.

**TABLE 2:** Materials description: Concrete (Schrefler et al. 2002) and asphalt mixture (Bonati et al. 2015).

	Tunnel walls and ceiling	Road pavement
**Material**	Concrete	Asphalt mixture
**Thickness**	0.5 m	0.4 m
**Density**	2585 kg/m^3^	2275 kg/m^3^
**Thermal conductivity**	1.67 W/ (m · K)	0.56 W/ (m · K)
**Specific heat**	0.94 kJ/ (kg · K)	0.88 kJ/ (kg · K)

### 3.2 Accident Scenario

The accident scenario consists of a release of LH_2_ from a 50‐mm diameter hole located at 0.5 m above the road surface on the back side of a road tanker assumed to be involved in a traffic collision. The incidental road tanker is schematized as a 12‐m long cylinder with a diameter of 2.2 m and is assumed to be positioned along the right driving lane of the tunnel tube in the middle of its length (i.e., 500 and 1000 m from the entrance portal of the 1 and 2 km long tunnel tube, respectively). The above‐mentioned longitudinal location of the road tanker responsible for the LH_2_ leak was selected among others because it was found to be the worst in terms of the size of the flammable hydrogen cloud (Caliendo et al. 2023). It is to be said that LH_2_ is typically transported within 45 m^3^ insulated road tankers that can contain up to 2500 kg of LH_2_ at its atmospheric boiling point (i.e., −253.15°C) and 4 bar pressure (Hall et al. 2014). Considering this, since the mass flow rate of LH_2_ from the hole was computed to be 7.7 kg/s and constant over time, the LH_2_ release lasts approximately 5 min before the tanker is empty.

### 3.3 Queue Formation and People Evacuation Process

**FIGURE 2:**
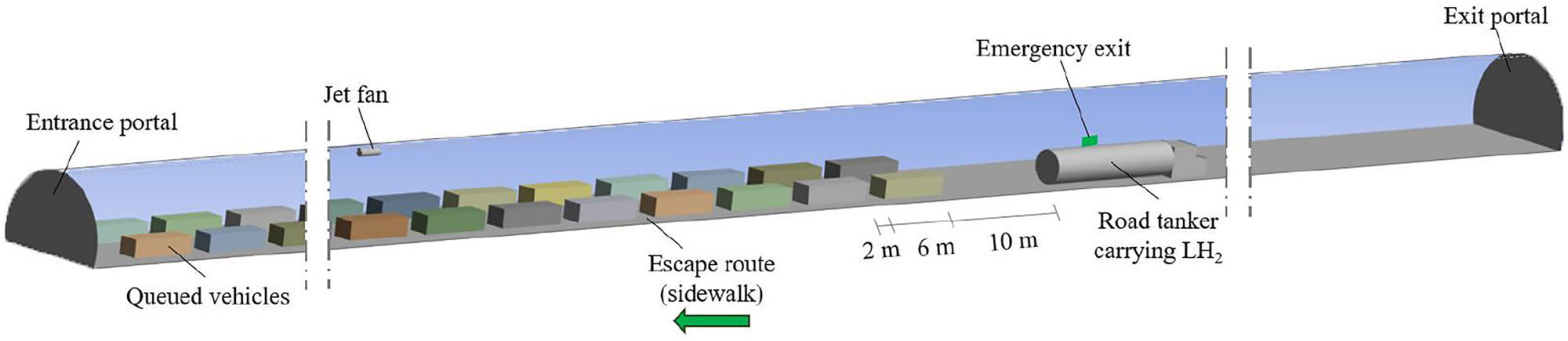
Schematization of the 1 km long unidirectional tunnel tube under consideration in the presence of a longitudinal mechanical ventilation system, with a view of the road tanker carrying LH_2_, the vehicles in the queue, the two portals, the emergency exit, and escape routes.

Once the above‐mentioned accident scenario occurs in the tube, the vehicles downstream of the road tanker responsible for the LH_2_ spill are assumed to leave the unidirectional tunnel tube under consideration using its exit portal; while those upstream of the LH_2_ release source, since they are considered to be unable to overtake the incidental road tanker for safety reasons, queue up along the centerline of both driving lanes maintaining a longitudinal space of 2 m from each other. Among the queued vehicles, the first one (i.e., the one closest to the LH_2_ release source) is taken to be located at a distance of 10 m from the back side of the incidental road tanker. To facilitate CFD analyses, it is also assumed that the queue of vehicles consists of equivalent vehicles (i.e., equivalent passenger cars), which are schematized in the simulations as parallelepipeds of 6 m in length, 1.8 m in width, and 1.5 m in height. Figure 2 shows the schematization of the 1 km long unidirectional tunnel tube under consideration in the presence of a longitudinal mechanical ventilation system, with a view of the road tanker carrying LH_2_, the vehicles in the queue, the two portals, the emergency exit, and escape routes (i.e. the two sidewalks).

Since the number of people potentially at risk is expected to increase as the number of queued vehicles increases, and thus to perform a more conservative analysis, the tunnel portion upstream of the incidental road tanker is considered to be full of stopped vehicles when the LH_2_ release starts. Considering these assumptions, the total number (i.e., including both driving lanes) of vehicles queued upstream of the LH_2_ release source situated in the middle of the tunnel is 120 and 246 for the 1 and 2 km long tunnel, respectively. By assuming an average occupancy rate of 2 people per equivalent vehicle (i.e., 1.7 people for passenger cars (Caliendo et al. 2018), 1 person for HGVs, and 30 people for buses), the total number of people trapped in the tunnel was found to be 240 or 492 for the 1 or 2 km long tunnels. Based on the different traffic volumes considered in the parametric analysis, the time (*t*) needed by all the vehicles to fulfill the tunnel portion upstream of the incidental road tanker was computed to be 196, 123, and 90 s for the 1 km long tunnel or 403, 253, and 185 s for the 2 km long tunnel when PHV is 1100, 1750, and 2400 vehicles/h per lane, respectively.

For all the users, the detection time, considered from the start of the LH_2_ release, is set to be equal to 60 s, as well as the reaction time (time to react to a hazardous situation and leave own vehicle stopped in the queue) is 30 s. At this pre‐movement time (i.e., 90 s), the time of each vehicle to enter the tunnel tube and stop in the queue must be added. In this respect, it is to be recorded that for the PHV of 2400, 1750, and 1100 vehicles/h per lane, the entering frequency of vehicles per unit time is on average 1.5, 2.1, and 3.3 s, respectively. This means, for example, that with reference to the tunnel length of 1 km and the PHV of 2400 vehicles/h per lane, after 60 s from the start of the release of LH_2_, there are already 40 vehicles per lane stopped in the queue upstream of the tanker, while the remaining 20 vehicles per lane enter the tunnel within the following 30 s. Considering a PHV of 2400 vehicles/h per lane, it is also to be mentioned that the first evacuee (i.e., the one closest to the incidental road tanker) will move 0.6 or 1.8 s earlier than the case related to the PHV of 1750 or 1100 vehicles/h per lane.

The average walking speed that all the users maintain along the escape routes (i.e., the two sidewalks) to reach a safe place was considered to be 0.5 m/s. Furthermore, in the case of the 1 km long tunnel, the evacuees can leave the structure only through the entrance portal of the tunnel since the emergency exit located halfway along the tunnel tube length, where the accident scenario is set to occur, is assumed to be inaccessible to the users for safety reasons. While, apart from the emergency exit situated near the incidental road tanker that was assumed to be inaccessible, the tunnel occupants are considered to exit the 2 km long tunnel tube using both the emergency exit located at 500 m from the entrance portal and this last one.

### 3.4 Research Framework

This study is set within the field of research on tunnel safety concerning the transport of LH_2_ by road tankers. The paper relies on the development of specific CFD models of LH_2_ release and explosion to assess the potential impacts on tunnel users. However, it extends the state‐of‐the‐art by carrying out a parametric analysis aimed at quantifying the entity of benefits due to mechanical ventilation on the tunnel safety in the event of LH_2_ release, compared to the natural one, as a function of geometry and traffic conditions. Given the lack of studies based on the combined effect of the above‐mentioned parameters, this paper aims to provide additional knowledge to the field of the risk analysis of the release of LH_2_ in road tunnels, and might also serve as a support tool for tunnel management agencies in the choice of mitigation measures and/or traffic control strategies to limit the negative consequences due this substance.

**FIGURE 3:**
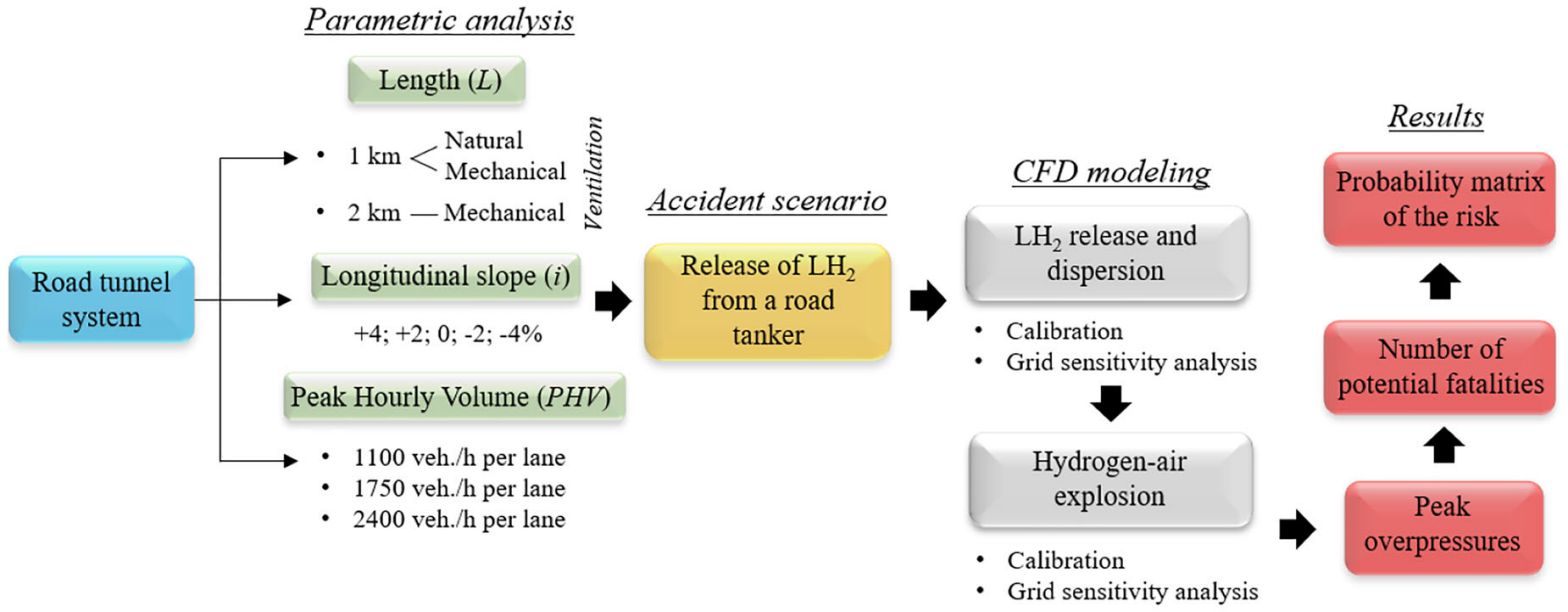
Flow chart of the methodology.

The steps followed to achieve the objectives outlined above are shown schematically in Figure 3.

## 4 CFD Modeling

### 4.1 Overview

The current software market offers several 3D CFD codes. Among those, the one applied in this study is ANSYS Fluent version 2023 R1 (ANSYS Fluent Theory Guide 2023), a widely recognized commercial CFD software, which is particularly suitable for simulating LH_2_ release and dispersion phenomena (Liu et al. 2018; Tang et al. 2020; Sun et al. 2024), as well as for reproducing explosion scenarios (Mao et al. 2021; T. Zhang et al. 2022; Hu et al. 2023). The ANSYS Fluent code numerically solves the Navier–Stokes equations, providing results, such as hydrogen concentrations and overpressures in case of ignition, with respect to each cell constituting the mesh used to discretize the computational domain.

### 4.2 Initial and Boundary Conditions

The ambient conditions inside the tunnel tube when the accidental scenario occurs were defined by setting the atmospheric pressure of 101,325 Pa and the temperature of 15°C (Liu et al. 2018). To simulate natural ventilation due to the piston effect of vehicles in motion, a positive pressure difference of +5 Pa was set between the two tunnel portals. In the case of mechanical ventilation, to reproduce jet fan action, each fan was modeled as a velocity inlet (maximum velocity of 30 m/s), activated 60 s after the LH_2_ release. This generated an average longitudinal airflow of ∼5 m/s. Moreover, no‐slip boundary conditions were assigned to both the tunnel walls and the road pavement (Molkov and Dery 2020). Finally, the mass flow inlet boundary condition was attributed to the surface of the hole through which LH_2_ leakage occurs.

### 4.3 LH_2_ Release and Dispersion

#### 4.3.1 Simulation Setup

To reproduce the whole flow process involving the release, dispersion, and evaporation of LH_2_ in the tunnel environment, the mixture model was implemented as a multiphase model (Shao et al. 2018; Liu et al. 2019; Sun et al. 2021). In this model, two main phases can be identified: the first is liquid and involves LH_2_, while the second is gaseous and consists of gas hydrogen and air. The phase change process from liquid to gas is reproduced by the evaporation–condensation Lee model, which regulates the mass transfer between different phases on the basis of the saturation temperature of hydrogen, which is set at −252.88°C (Verfondern and Dienhart 2007). Since the Mach number (i.e., the ratio between the flow velocity and the speed of sound) of the flow in the computational domain is ≤ 0.3, all the gases were set as incompressible for the release simulations.

The *k*–*ε* sub model of the Reynolds Averaged Navier–Stokes (RANS) turbulence model, which separately solves the two transport equations concerning turbulent kinetic energy (*k*) and turbulent dissipation rate (*ε*), was used to simulate the turbulence flows involving LH_2_ releases and dispersion (Shao et al. 2018; Liu et al. 2019; Sun et al. 2021).

The Pressure‐Implicit with a Splitting of Operators (PISO) scheme was selected for pressure–velocity coupling, while a second order upwind scheme was used to solve the turbulent kinetic energy and turbulent dissipation rate (Sun et al. 2024). The solution was assumed to be converged when the residual was less than 10^−3^; whereas the time step of the simulations was set at 10^−3^ s, which was found to be a good compromise between accuracy and computational time.

#### 4.3.2 CFD Calibration Against LH_2_ Release

**FIGURE 4:**
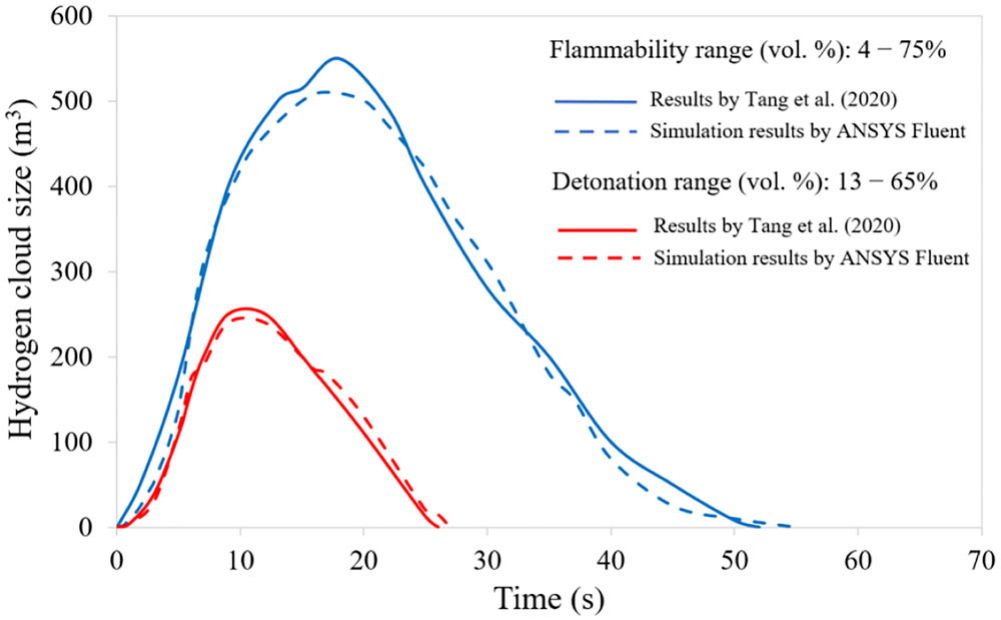
Comparison of the hydrogen cloud size within the flammability and detonation range between the results by Tang et al. (2020) and the simulated ones by ANSYS Fluent.

To calibrate the CFD code with reference to LH_2_ release in confined spaces, a numerical study (Tang et al. 2020) involving the release of 1 kg/s of LH_2_ in a real size road tunnel was replicated with ANSYS Fluent. In our paper, the simulated hydrogen cloud size both within the flammability range (i.e., between 4% and 75% in air) and detonation range (i.e., between 13% and 65% in air) was compared to that obtained by Tang et al. (2020), and an error lower than 5% was always found (Figure 4). Based on this calibration, the 3D CFD model involving the release and dispersion of LH_2_ inside the full‐scale road tunnel under investigation was subsequently developed.

#### 4.3.3 Discretization of the Computational Domain

**FIGURE 5:**
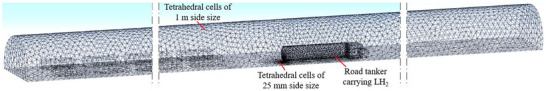
Discretization of the computational domain into tetrahedral cells, with a mesh refinement around the hole through which liquid hydrogen leakage occurs.

A very fine mesh consisting of tetrahedral cells with 25 mm side size was adopted to discretize the computational domain near the LH_2_ release source (Molkov and Dery 2020), while coarser elements were used along the remaining part of the tunnel to reduce the calculation time. However, to ensure that the dimension of coarser cells was not too large to lead to inaccurate predictions, a grid sensitivity analysis was made. Regarding the case of the 1 km long tunnel, the results of the grid sensitivity analysis showed that tetrahedral cell sizes smaller than 1 m did not significantly improve the accuracy of model predictions, even though they required much more calculation time. When comparing the results obtained using cells with dimensions of 0.5 and 1 m, the difference in the simulated hydrogen concentration was always found to be lower than 5%. Figure 5 shows the discretization of the computational volume into tetrahedral elements of 1 m side size, with a grid cell refinement up to 25 mm around the hole through which LH_2_ leakage occurs. With reference to the 2 km long tunnel tube, the calculation domain defined for the 1 km long tunnel tube was extended (using cells of 1 m side size) by adding a 500 m portion both upstream and downstream of its entrance and exit portals, respectively. As a result, a total of 454,550 or 865,674 tetrahedral cells were obtained for the 1 or 2 km long tunnels.

### 4.4 Hydrogen‐Air Explosion

#### 4.4.1 Simulation Configuration

To simulate explosion scenarios in ANSYS Fluent, it is necessary to define the sub‐models regulating the processes of combustion, turbulence, and thermal radiation. These sub‐models are incorporated into the compressible three‐dimensional transient equations of continuity, momentum, and energy of a mixture of ideal gases (T. Zhang et al. 2022).

As far as the combustion model is concerned, the species transport equation was used to solve the conservation equations describing convection, diffusion, and reaction sources for each species contained within the hydrogen‐air mixture (T. Zhang et al. 2022; Goswami and Sun 2022).

Large Eddy Simulation (LES) turbulence approach was chosen for its suitability to transient flows and blast waves (Molkov and Dery 2020; T. Zhang et al. 2022), while subgrid‐scale stresses were solved by the Wall‐Adapting Local Eddy‐viscosity (WALE) subgrid‐scale model (T. Zhang et al. 2022).

Moreover, no thermal radiation model was considered in this study since hydrogen–air explosions are so rapid that the generated heat can be neglected (Qin and Chen 2021; Y. Li et al. 2021).

The pressure‐velocity coupling was solved by the SIMPLEC solution method (Qin and Chen 2021; Shen et al. 2021), with convergence conditions considered to be reached when solution residuals drop below 10^−3^. A time step smaller than that used for the LH_2_ release scenarios was chosen for the explosion simulations (i.e., 10^−5^vs. 10^−3^ s) as they last only a few seconds, thus requiring a higher temporal discretization (Tran et al. 2018; Goswami and Sun 2022, G. Li et al. 2022).

To simulate the hydrogen–air explosions in ANSYS Fluent, the environmental conditions within the tunnel in terms of the spatial distribution of the hydrogen cloud at the time of ignition must be recreated, the location and spark ignition energy defined, and the sub‐models describing combustion and turbulence specified.

#### 4.4.2 CFD Calibration Against Hydrogen Explosion

**FIGURE 6:**
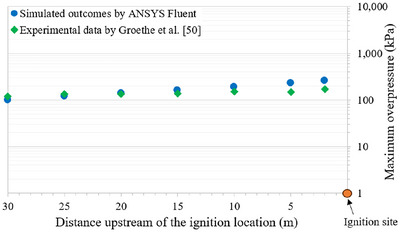
Comparison of the maximum overpressure predicted by ANSYS Fluent and those obtained experimentally by Groethe et al. (2007) at 1 m height along the tunnel centreline upstream of the ignition point.

An experimental test performed by Groethe et al. (2007) was replicated using ANSYS Fluent to calibrate it against actual scenarios involving hydrogen explosions in confined spaces. The computational domain was discretized into tetrahedral cells of 0.3 m side size (Molkov et al. 2008), for a total of 73,377 elements. By comparing the maximum overpressures at 1 m height along the tunnel centerline upstream of the ignition location (Figure 6), a good agreement was found between the simulated and experimental results.

In the light of the above‐mentioned calibration process, the 3D CFD model involving a hydrogen–air explosion within the investigated full‐scale road tunnel after the LH_2_ leakage was set up.

#### 4.4.3 Discretization of the Calculation Volume

**FIGURE 7:**
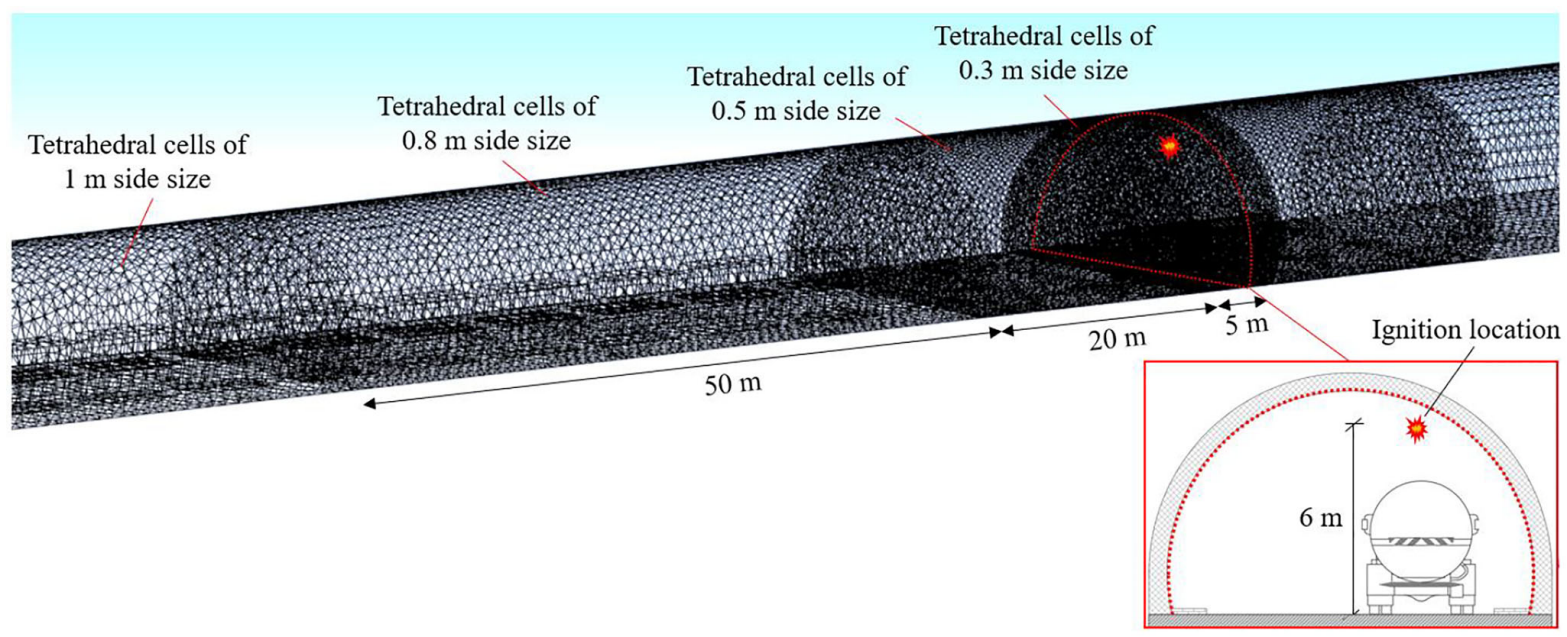
Discretization of the tunnel volume into tetrahedral elements, with a gradual mesh refinement towards the ignition point.

The optimal mesh resolution (i.e., the cell size representative of a good compromise between the accuracy of the model predictions and the computational time) for the explosion simulations was identified by carrying out a new grid sensitivity analysis. By considering the case of the 1 km long tunnel, the results of the grid sensitivity analysis led to a refined mesh near ignition (0.3–0.8 m) and coarser cells (1 m) elsewhere as shown in Figure 7. The aforementioned cell discretization was also used for the 2 km long tunnel tube, where its exceeding length compared to that of the 1 km long tunnel (i.e., 500 m both upstream and downstream of the entrance and exit portals, respectively) was subdivided into cells of 1 m side size. Considering this, a total of 820,961 and 1,152,040 tetrahedral elements were used for the 1 and 2 km long tunnels, respectively.

As also shown in Figure 7, the flammable hydrogen‐air mixture was assumed to be ignited under the tunnel ceiling (i.e., 6 m above the road surface) in the middle of the tunnel length (i.e., 500 and 1000 m from the entrance portal of the 1 and 2 km long tunnel tube, respectively) by a 0.1 J electrical spark (Y. Zhang et al. 2019). At that height, electrical equipment (e.g., tunnel lighting systems) may likely ignite the flammable hydrogen cloud developed after the accidental release of LH_2_ (Venetsanos et al. 2008).

## 5 Analysis of the Results

### 5.1 LH_2_ Release

Once released, LH_2_ rapidly evaporates due to the extremely low temperature at which it is stored inside the tank and, consequently, accumulates beneath the tunnel ceiling leading to the formation of a highly flammable hydrogen cloud. In this paper, dispersion simulations were conducted over a 10‐min period to account for evacuation time of people from the tunnel tube, while the LH_2_ release lasted around 5 min, which is the time required to empty the road tanker based on both its capacity and LH_2_ mass flow rate.

The CFD results in terms of size of the flammable hydrogen cloud presented below refer to the time instant at which the tunnel under investigation is filled with vehicles (i.e., 90, 123, and 196 s for the 1 km long tunnel or 185, 253, and 403 s for the 2 km long tunnel when PHV is 2400, 1750, and 1100 vehicles/h per lane, respectively).

#### 5.1.1 Flammable Hydrogen Cloud in the 1 Km Long Tunnel Tube Under Natural Ventilation

**FIGURE 8:**
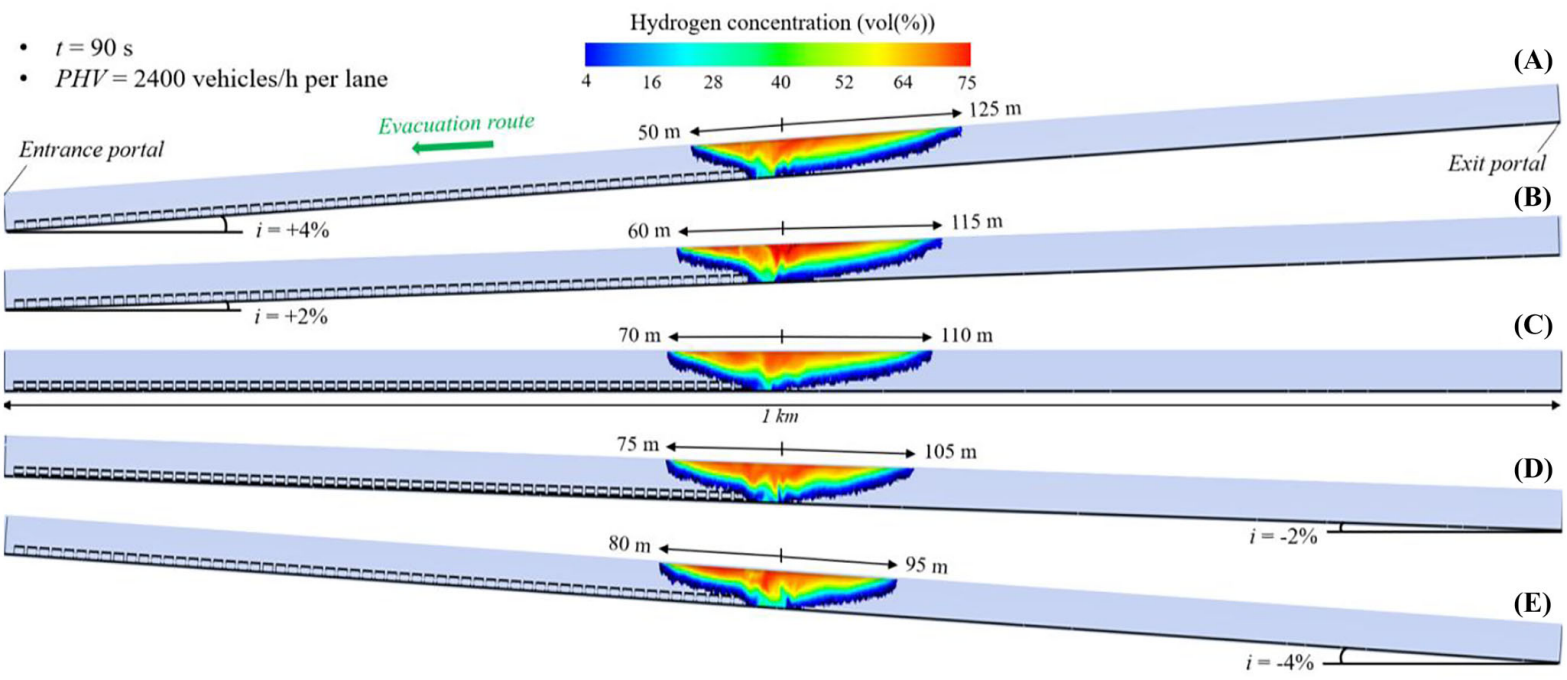
Flammable hydrogen cloud within the 1 km long tunnel tube under natural ventilation after 90 s (PHV = 2400 vehicles/h per lane) from the LH_2_ release for a longitudinal slope of: (a) *i* = +4%, (b) *i* = +2%, (c) *i* = 0, (d) *i* = −2%, (e) *i* = −4%.

**FIGURE 9:**
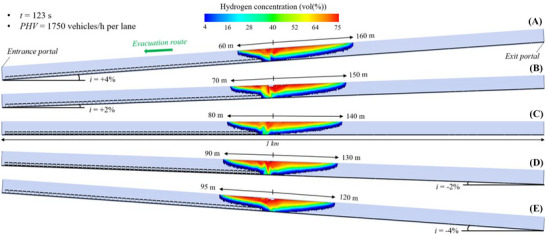
Flammable hydrogen cloud within the 1 km long tunnel tube under natural ventilation after 123 s (PHV = 1750 vehicles/h per lane) from the LH_2_ release for a longitudinal slope of: (a) *i* = +4%, (b) *i* = +2%, (c) *i* = 0, (d) *i* = −2%, (e) *i* = −4%.

**FIGURE 10:**
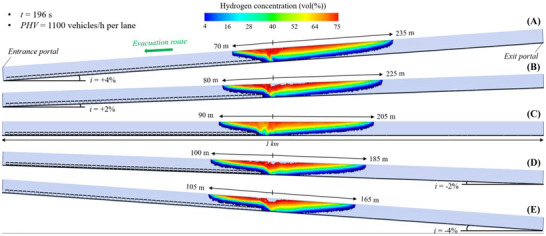
Flammable hydrogen cloud within the 1 km long tunnel tube under natural ventilation after 196 s (PHV = 1100 vehicles/h per lane) from the LH_2_ release for a longitudinal slope of: (a) *i* = +4%, (b) *i* = +2%, (c) *i* = 0, (d) *i* = −2%, (e) *i* = −4%.

Figure 8, Figure 9, and Figure 10 depict the spatial distribution of the hydrogen cloud between its flammability limits (i.e., from 4% to 75% in air) within the 1 km long naturally ventilated tunnel tube considered to have a longitudinal slope of +4, +2, 0, −2, or −4% after 90 s (PHV = 2400 vehicles/h per lane), 123 s (PHV = 1750 vehicles/h per lane), and 196 s (PHV = 1100 vehicles/h per lane) from when the LH_2_ release starts, respectively. In general, higher hydrogen concentrations are found beneath the tunnel ceiling because of the high buoyancy of hydrogen that tends to rise upwards once released. Moreover, the propagation of the hydrogen–air mixture upstream and downstream of the spill site is not symmetrical due to the effects of both the natural ventilation pushing the hydrogen cloud in the direction of the exit portal of the tunnel and the longitudinal slope driving the hydrogen–air mixture towards the exit or entrance portal of the tunnel in the case of positive (i.e., the chimney effect) or negative (i.e., the inverse chimney effect) gradients.

By observing each of the above‐mentioned figures, it is worth noting that the distance reached by the flammable hydrogen cloud upstream of the release source increases as the longitudinal slope becomes negative (i.e., passing from +4% to −4%). This is caused by the progressively greater contribution of the inverse chimney effect in pushing the hydrogen–air mixture more and more in the opposite direction of the natural ventilation (i.e., towards the entrance portal of the tunnel tube where the escaping users are headed). Specifically, passing from *i* = +4% to *i* = −4%, the longitudinal extension of the flammable hydrogen cloud upstream of the leakage point was found to be between 50 and 80 m (Figure 8), between 60 and 95 m (Figure 9), and between 70 and 105 m (Figure 10) after 90, 123, and 196 s from the start of the LH_2_ release, respectively. On the contrary, the distance covered by the hydrogen–air mixture downstream of the spill location reduces passing from *i* = +4% to *i* = −4%, ranging from 125 to 95 m, from 160 to 120 m, and from 235 to 165 m after 90, 123, and 196 s since the LH_2_ spill start, respectively.

By comparing Figure 8, Figure 9, and Figure 10, it is also possible to note that under the same value of the longitudinal slope, the spatial extension of the flammable hydrogen cloud both upstream and downstream of the leakage site increases in the time range between 90 and 196 s since the amount of LH_2_ released increases over time until the tanker is emptied, which happens at *t* = 300 s from when the LH_2_ leak starts.

#### 5.1.2 Flammable Hydrogen Cloud in the 1 Km‐Long Tunnel Tube Under Mechanical Ventilation

**FIGURE 11:**
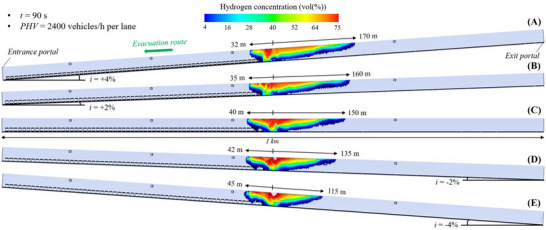
Flammable hydrogen cloud within the 1 km long tunnel tube under longitudinal mechanical ventilation after 90 s (PHV = 2400 vehicles/h per lane) from the LH_2_ release for a longitudinal slope of: (a) *i* = +4%, (b) *i* = +2%, (c) *i* = 0, (d) *i* = −2%, (e) *i* = −4%.

**FIGURE 12:**
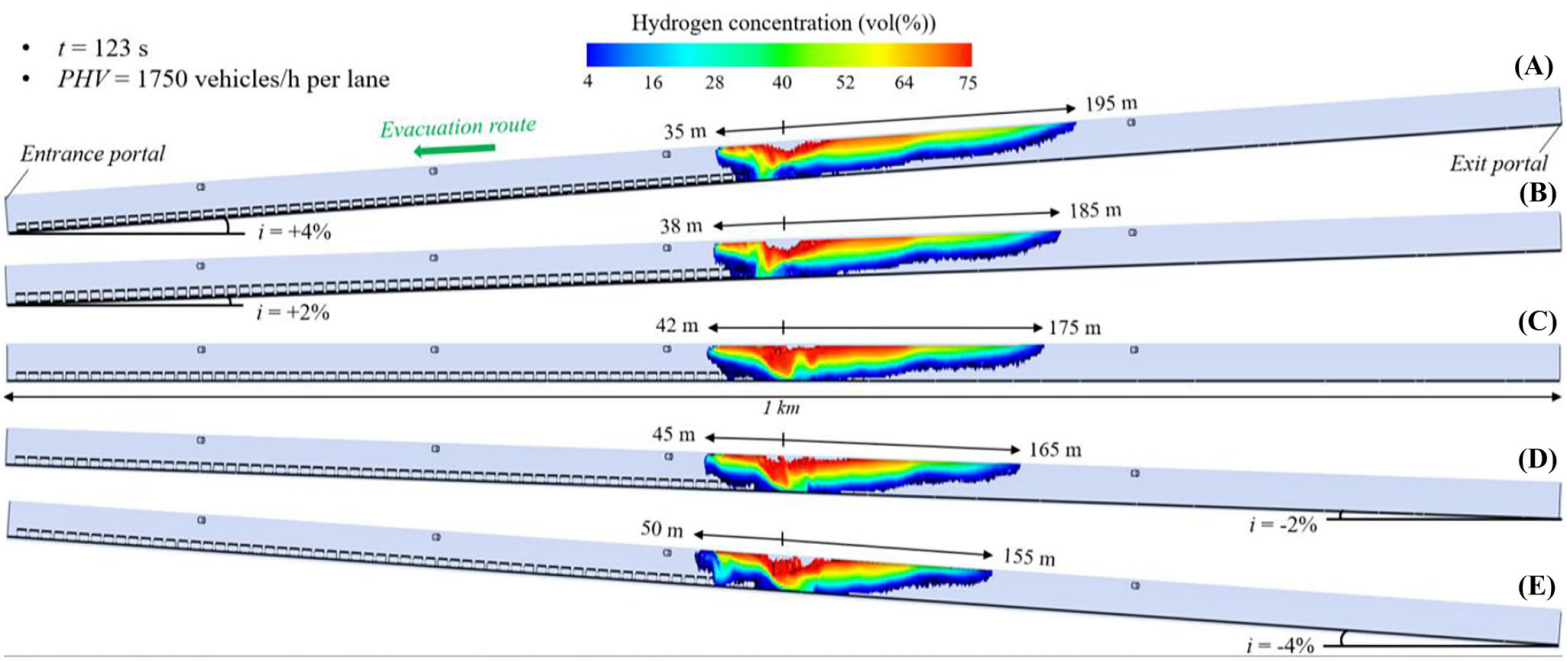
Flammable hydrogen cloud within the 1 km long tunnel tube under longitudinal mechanical ventilation after 123 s (PHV = 1750 vehicles/h per lane) from the LH_2_ release for a longitudinal slope of: (a) *i* = +4%, (b) *i* = +2%, (c) *i* = 0, (d) *i* = −2%, (e) *i* = −4%.

**FIGURE 13:**
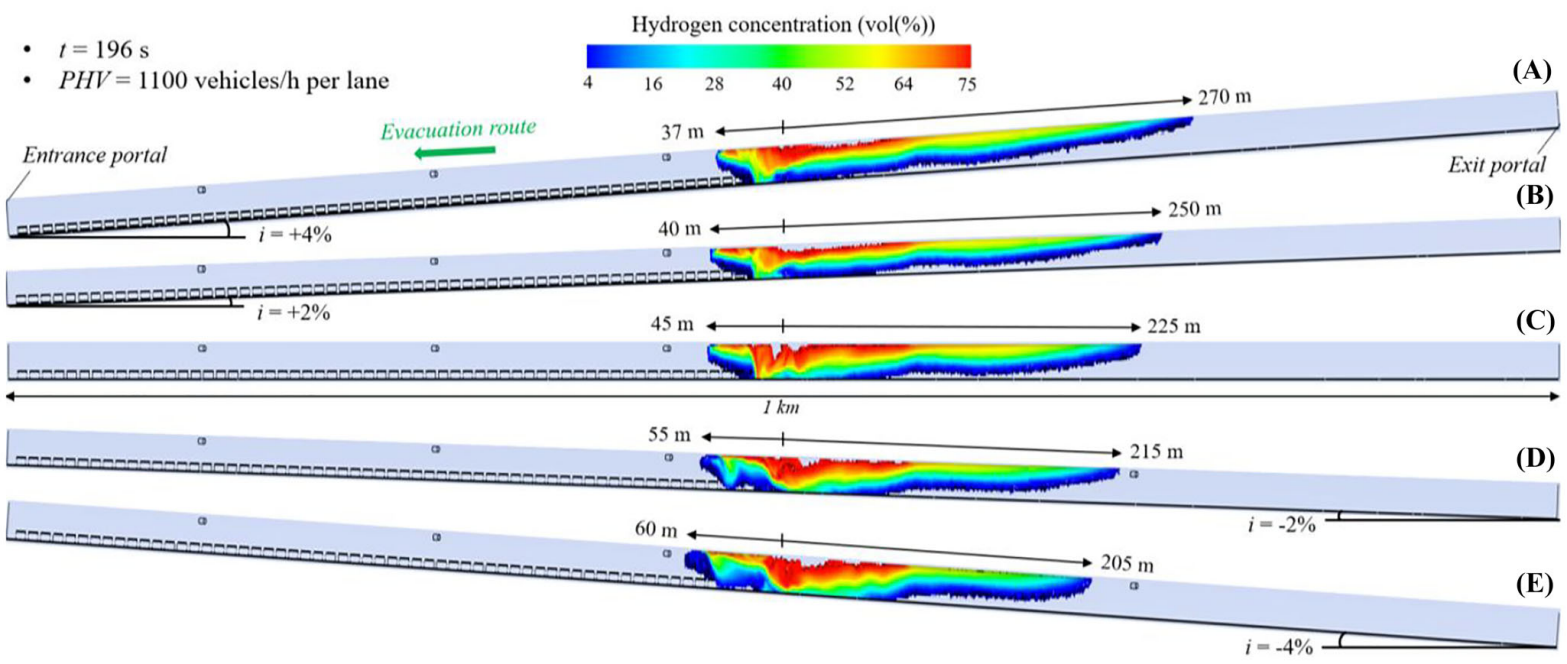
Flammable hydrogen cloud within the 1 km long tunnel tube under longitudinal mechanical ventilation after 196 s (PHV = 1100 vehicles/h per lane) from the LH_2_ release for a longitudinal slope of: (a) *i* = +4%, (b) *i* = +2%, (c) *i* = 0, (d) *i* = −2%, (e) *i* = −4%.

For each value of the longitudinal slope (i.e., +4, +2, 0, −2, or −4%), Figure 11, Figure 12, and Figure 13 show the spatial distribution of the flammable hydrogen cloud within the 1 km long tunnel tube, which is assumed in this case to be mechanically ventilated, after a time of 90 s (PHV = 2400 vehicles/h per lane), 123 s (PHV = 1750 vehicles/h per lane), and 196 s (PHV = 1100 vehicles/h per lane) from the start of the LH_2_ release, respectively. It is possible to note that the vertical stratification of the hydrogen concentration along the structure is significatively affected by the type of ventilation. The activation of the jet fans leads to a higher reduction in the average value of the hydrogen concentration when compared with natural ventilation condition (i.e., for the same longitudinal slope and time from the start of the LH_2_ release, the red area reported in Figure 13a is smaller than that of Figure 10a). Furthermore, in the presence of a longitudinal mechanical ventilation system, the spatial extension of the flammable hydrogen cloud downstream of the release location is much longer than in the case of natural ventilation due to the jet fans that push the air flow towards the exit portal of the tunnel tube. Consequently, a non‐symmetrical spread of the flammable hydrogen cloud upstream and downstream of the LH_2_ release center is found. On the contrary, the propagation of the flammable hydrogen cloud upstream of the spill location (i.e., according to direction of users’ evacuation) is much more contained when the tunnel tube is mechanically instead of naturally ventilated.

It is also to be noted that when the tunnel is mechanically ventilated, longer distances of the hydrogen–air mixture upstream of the leakage point are reached under negative rather than positive gradients. Passing from *i* = +4% to *i* = −4%, the longitudinal extension of the propagation of the flammable hydrogen upstream of the spill site was found to range between 32 and 45 m (Figure 11), between 35 and 50 m (Figure 12), and between 37 and 60 m (Figure 13) after 90, 123, and 196 s from the start of the LH_2_ release, respectively. This means that more users escaping towards the entrance portal of the tunnel tube might be involved by the propagation of the hydrogen cloud.

#### 5.1.3 Flammable Hydrogen Cloud in the 2 Km Long Tunnel Tube Under Mechanical Ventilation

**FIGURE 14:**
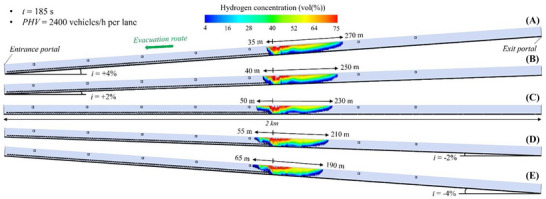
Flammable hydrogen cloud within the 2 km long tunnel tube under longitudinal mechanical ventilation after 185 s (PHV = 2400 vehicles/h per lane) from the LH_2_ release for a longitudinal slope of: (a) *i* = +4%, (b) *i* = +2%, (c) *i* = 0, (d) *i* = −2%, (e) *i* = −4%.

**FIGURE 15:**
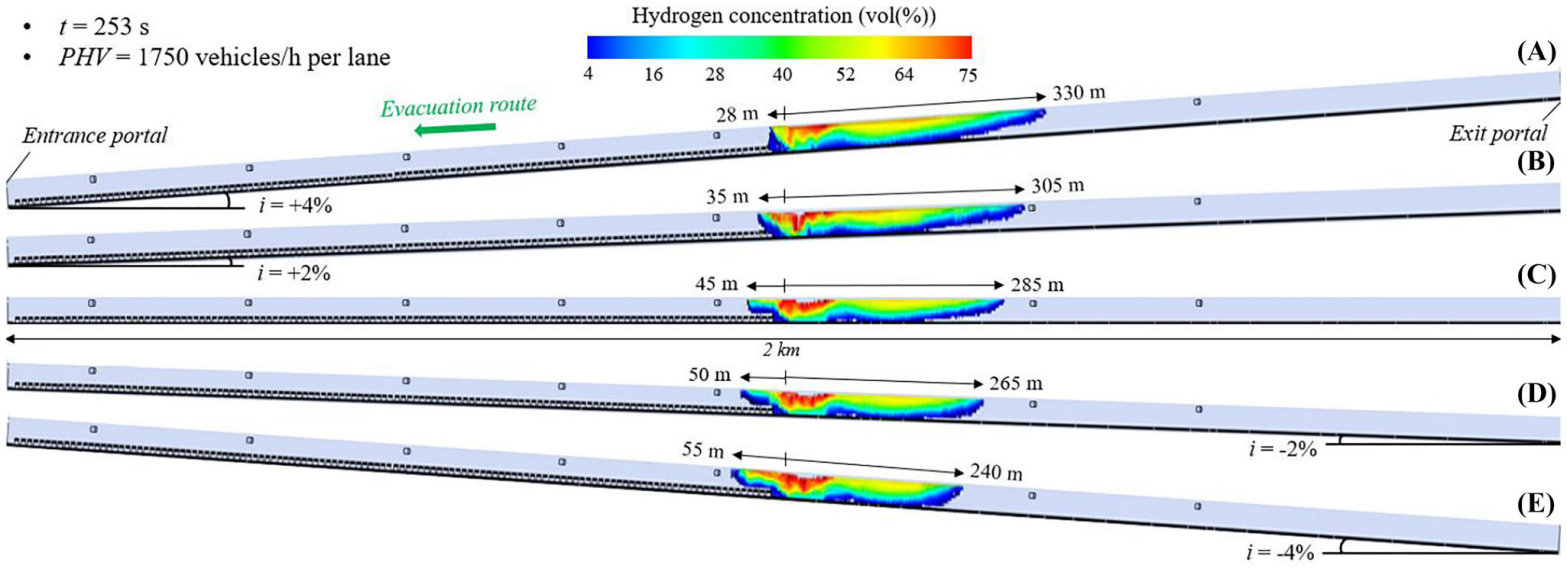
Flammable hydrogen cloud within the 2 km long tunnel tube under longitudinal mechanical ventilation after 253 s (PHV = 1750 vehicles/h per lane) from the LH_2_ release for a longitudinal slope of: (a) *i* = +4%, (b) *i* = +2%, (c) *i* = 0, (d) *i* = −2%, (e) *i* = −4%.

**FIGURE 16:**
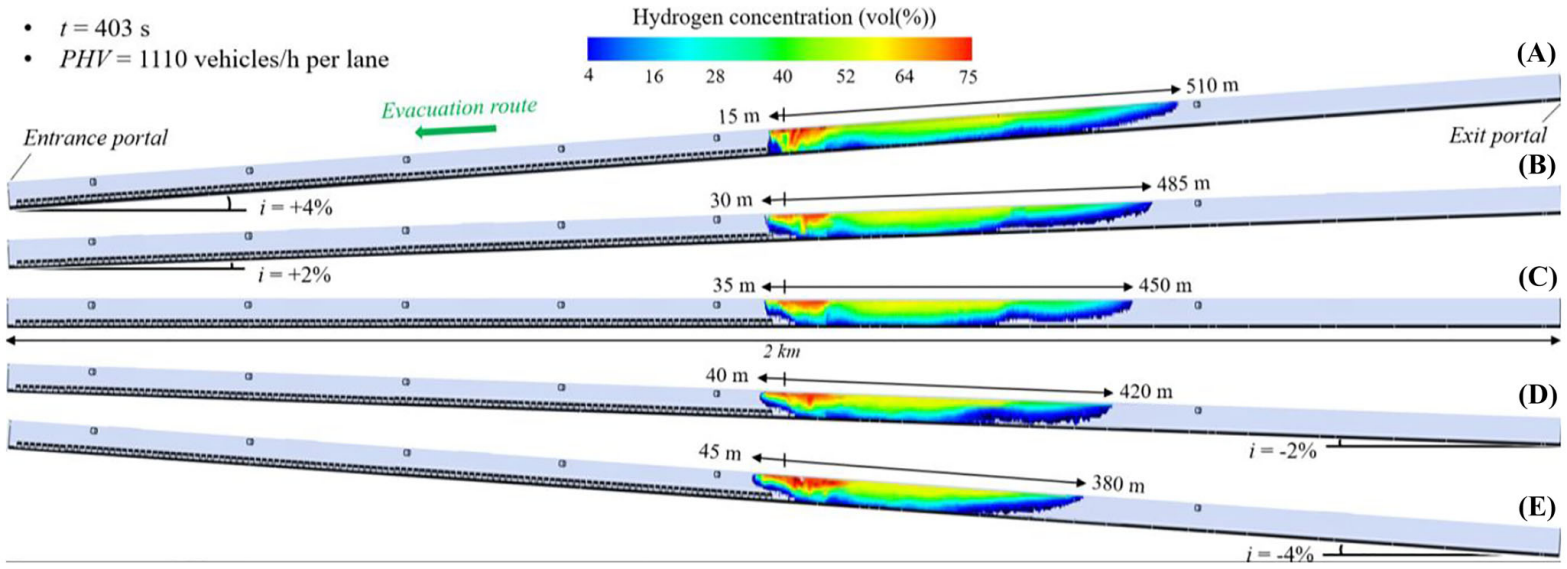
Flammable hydrogen cloud within the 2 km long tunnel tube under longitudinal mechanical ventilation after 403 s (PHV = 1100 vehicles/h per lane) from the LH_2_ release for a longitudinal slope of: (a) *i* = +4%, (b) *i* = +2%, (c) *i* = 0, (d) *i* = −2%, (e) *i* = −4%.

Figure 14, Figure 15, and Figure 16 show the spatial distribution of the flammable hydrogen cloud along the 2 km long tunnel tube, which is always assumed to be mechanically ventilated, for inclinations of +4, +2, 0, −2, or −4%, and times of 185 s (PHV = 2400 vehicles/h per lane), 253 s (PHV = 1750 vehicles/h per lane), and 403 s (PHV = 1100 vehicles/h per lane) from the start of the LH_2_ release, respectively. Each figure confirms that the distance reached by the hydrogen–air mixture upstream of the leakage site increases passing from positive to negative gradients, while decreasing downstream. Passing from *i* = +4% to *i* = −4%, the hydrogen–air mixture extends upstream of the spill location for a distance between 35 and 65 m (Figure 14), between 28 and 55 m (Figure 15), and between 15 and 45 m (Figure 16) after 185, 253, and 403 s from the start of the LH_2_ release, respectively. Regarding the shorter distance covered by the flammable hydrogen cloud upstream of the leakage point after 253 compared to 185 s from when the LH_2_ spill occurs (Figure 15 vs. Figure 14), this may be due to the increase over time of the air flow velocity along the tube generated by the jet fans. We found that the velocity of the air flow towards the exit portal (i.e., downstream of the hydrogen release) increased over time passing from 185 and 253 s, reaching steady‐state conditions of 5 m/s after approximately 220 s from the release start. The further reduction in the longitudinal extension of the flammable hydrogen cloud upstream of the leakage point at 403 s compared to 253 s (Figure 16 vs. Figure 15) may be attributable to the fact that the release of LH_2_ ceased after 300 s from the start of the LH_2_ release (i.e., after this time the tank emptied). This might also explain why the extension of the red area (i.e., the one with the highest hydrogen concentration in the mixture) significantly decreased at the time of 403 s compared to 253 s.

In the light of the above considerations, by comparing Figure 14, Figure 15, and Figure 16 for a given gradient, it is found that the propagation of the hydrogen–air mixture downstream of the spill point increases over time.

Table 3 summarizes the CFD results reported above in terms of the spatial distribution of the flammable hydrogen cloud upstream of the LH_2_ release point (i.e., where the evacuation people are located) as a function of the tunnel length (*L*), type of ventilation (i.e., natural or longitudinal mechanical), time from the start of the LH_2_ release, and longitudinal slope (*i*).

**TABLE 3:** Distance reached by the flammable hydrogen cloud upstream of the LH_2_ release location as a function of the tunnel length (*L*), type of ventilation (i.e., natural or longitudinal mechanical), time from the start of the LH_2_ release, and longitudinal slope (*i*).

	Distance reached by the flammable hydrogen cloud upstream of the LH_2_ release location								
	*L* = 1 km						*L* = 2 km		
Type of ventilation	Natural			Longitudinal mechanical			Longitudinal mechanical		
**Time from the start of the LH_2_ release (s)**	90	123	196	90	123	196	185	253	403
**PHV (vehicles/h per lane)**	2400	1750	1100	2400	1750	1100	2400	1750	1100
***i* = −4%**	80 m	95 m	105 m	45 m	50 m	60 m	65 m	55 m	45 m
***i* = −2%**	75 m	90 m	100 m	42 m	45 m	55 m	55 m	50 m	40 m
***i* = 0**	70 m	80 m	90 m	40 m	42 m	45 m	50 m	45 m	35 m
***i* = +2%**	60 m	70 m	80 m	35 m	38 m	40 m	40 m	35 m	30 m
***i* = +4%**	50 m	60 m	70 m	32 m	35 m	37 m	35 m	28 m	15 m

It is possible to note that for the tunnel length of 1 km, for example, the distance reached by the hydrogen‐air mixture upstream of the spill site decreases with mechanical ventilation compared to the natural one. For the same time from the start of the LH_2_ release (i.e. 90, 123, or 196 s), or in other terms for the same PHV (i.e., PHV = 2400, 1750, or 1100 vehicles/h per lane), the aforementioned spatial distance also decreases as the longitudinal slope (*i*) becomes positive (i.e., from −4 to +4%) due to the chimney effect.

Moreover, as the tunnel length increases (i.e., *L* = 2 km against *L* = 1 km) under the longitudinal mechanical ventilation conditions, since the time considered from the start of the LH_2_ release is higher, which is assumed to be coincident with the time to fill the middle tunnel upstream of the LH_2_ leakage position (i.e., 185vs. 90 s for PHV = 2400 vehicles/h per lane, and 253vs. 123 s for PHV = 1750 vehicles/h per lane), the distance reached by the hydrogen–air mixture upstream of the spill location is longer for gradients between −4% and 0 (i.e., it is more significant the inverse chimney effect in the longer tunnel); while it is more or less similar or slightly lower for gradients between +2% and +4%. Finally, when the time considered from the start of the LH_2_ release is significantly higher (i.e., the time to fill the middle tunnel upstream of the LH_2_ leakage point is 403vs. 196 s for PHV = 1100 vehicles/h per lane), the distance reached by the hydrogen–air mixture upstream of the release location is shorter for all the gradients investigated, which is due to the increasing effect of the mechanical ventilation over time that pushes the hydrogen cloud towards the exit portal of the tunnel tube.

### 5.2 Hydrogen‐Air Mixture Explosion

Once released, the flammable hydrogen cloud might ignite generating overpressures inside the structure whose peak values along the right sidewalk (i.e., the closer escape route to the LH_2_ road tanker, see Figure 1) at breathing height (i.e., 1.90 m above the walking surface, see Point *A* of Figure 1) are reported below for all the cases investigated. The magnitude of overpressures due to the hydrogen–air mixture explosion depends on several factors, among which there are the ignition time of the flammable hydrogen cloud and its distribution along the structure, as well as the mass of hydrogen contained in the mixture with air at the time of ignition.

Concerning the ignition time, it is worth mentioning that there is no universally accepted reference in the current literature since the flammable hydrogen cloud might ignite at any moment following an accidental leak, also due to its extremely low ignition energy (i.e., 1.7 × 10^−5^ J). In this respect, for both the 1 km long naturally or mechanically ventilated tunnel and for the 2 km long mechanically ventilated tunnel, the ignition was assumed conservatively to occur after only 90 s from the LH_2_ release (Caliendo et al. 2024). In other terms, the mentioned ignition time was assumed to be equal to the value of the pre‐movement time of users (this last one is given for all users by the sum of the detection time [60 s] and reaction time [30s]).

Regarding the mass of hydrogen present in the cloud at the time of ignition, in this study only the mass of hydrogen at stoichiometric concentration in air (i.e., 29.5%) was considered to explode (Middha and Hansen 2009).

The explosion simulations were carried out for a relatively short time (i.e., approximately 4 and 8 s for the 1 and 2 km long tunnels, respectively), which is the time needed for the pressure wave to arrive at the entrance portal of the tunnel tube once the hydrogen–air mixture is ignited.

#### 5.2.1 Peak Overpressure Decay Along the 1 Km Long Tunnel Tube Under Natural Ventilation

**FIGURE 17:**
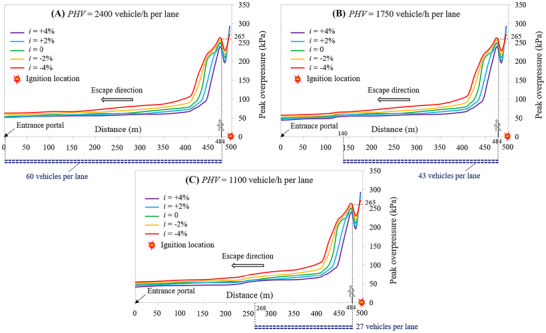
Peak overpressure decay upstream of the ignition location at the breathing height along the escape route of the 1 km long naturally ventilated tunnel tube as a function of the longitudinal slope for different peak hourly volumes: (a) PHV = 2400 vehicles/h per lane; (b) PHV = 1750 vehicles/h per lane; (c) PHV = 1100 vehicles/h per lane.

Assuming that the hydrogen cloud is ignited after 90 s from the LH_2_ release, Figure 17a−c depicts the peak overpressure decay upstream of the ignition location along the escape route of the 1 km long naturally ventilated tunnel tube at the breathing height as a function of the longitudinal slope (i.e., *i* = +4, +2, 0, −2, or −4%) for a PHV of 2400, 1750, and 1100 vehicles/h per lane, respectively. In this respect, it is possible to note that the overpressure reaches its highest value in the middle of the tunnel length where the ignition of the flammable hydrogen cloud was assumed to occur, then gradually decreasing towards the entrance portal of the tunnel at which it achieves its lowest value, although some oscillations can be observed due to its initial reflection with both the queued vehicles and tunnel structure (Molkov and Dery 2020).

For a given PHV, the region with the highest overpressure extends further upstream of the ignition location as the longitudinal slope of the tunnel tube decreases (i.e., passing from +4 to −4%, in other terms passing from the violet curve to the red one of Figure 17a−c). This means that the tunnel occupants are exposed along their escape route to higher overpressures as the gradient becomes negative. This result is coherent with that previously reported in terms of spatial distribution of the flammable hydrogen cloud, which was found to extend in the direction of the entrance portal of the tunnel for a distance that increased as the inclination dropped below zero.

From the mentioned figure, we can also see the position occupied by the last user leaving the structure (i.e., the one closest to the road tanker when the LH_2_ spill starts) at the time when the explosion of the hydrogen–air mixture occurs. Specifically, since the ignition time was assumed to be equal to the pre‐movement time of the users, the mentioned evacuee is still in its initial position next to its own car (i.e., 484 m from the entrance portal of the tunnel tube) when the explosion occurs, and he/she is subjected to a maximum overpressure of 230 kPa for *i* = +4%, which increases to 265 kPa when *i* = −4% for all the PHV*s* considered.

However, it is to be recorded that if the PHV decreases, the number of vehicles stopped in the queue at the moment of the ignition (i.e., 90 s after the release of LH_2_) is smaller passing from 60 to 43 up to 27 vehicles (i.e., corresponding to a PHV of 2400, 1750, and 1100 vehicles/h per lane, respectively). In this respect, Figure 17a shows that for the PHV = 2400 vehicles/h per lane the half of the tunnel tube upstream of the ignition (i.e., 485 m) is full of queued vehicles, while for PHV = 1750 and 1100 vehicles/h per lane, the length of the portion of the tunnel tube upstream of the ignition that is occupied by the vehicles stopped in the queue is 344 and 216 m, respectively (see Figure 17b–c). This may in theory mean that a greater number of users might be exposed to the risk of consequences due to an explosion when the traffic level is higher (i.e., 240, 172, and 108 people when the PHV is 2400, 1750, and 1100 vehicles/h per lane, respectively). The highest overpressures were found (see Figure 17a–c) to be located within a zone near the ignition point, the length of which is less than the aforementioned lengths of queued vehicles; as a result, the number of people having a high potential for death can be, in the case investigated, the same independent of the traffic level.

#### 5.2.2 Peak Overpressure Decay Along the 1 Km Long Tunnel Tube Under Mechanical Ventilation

**FIGURE 18:**
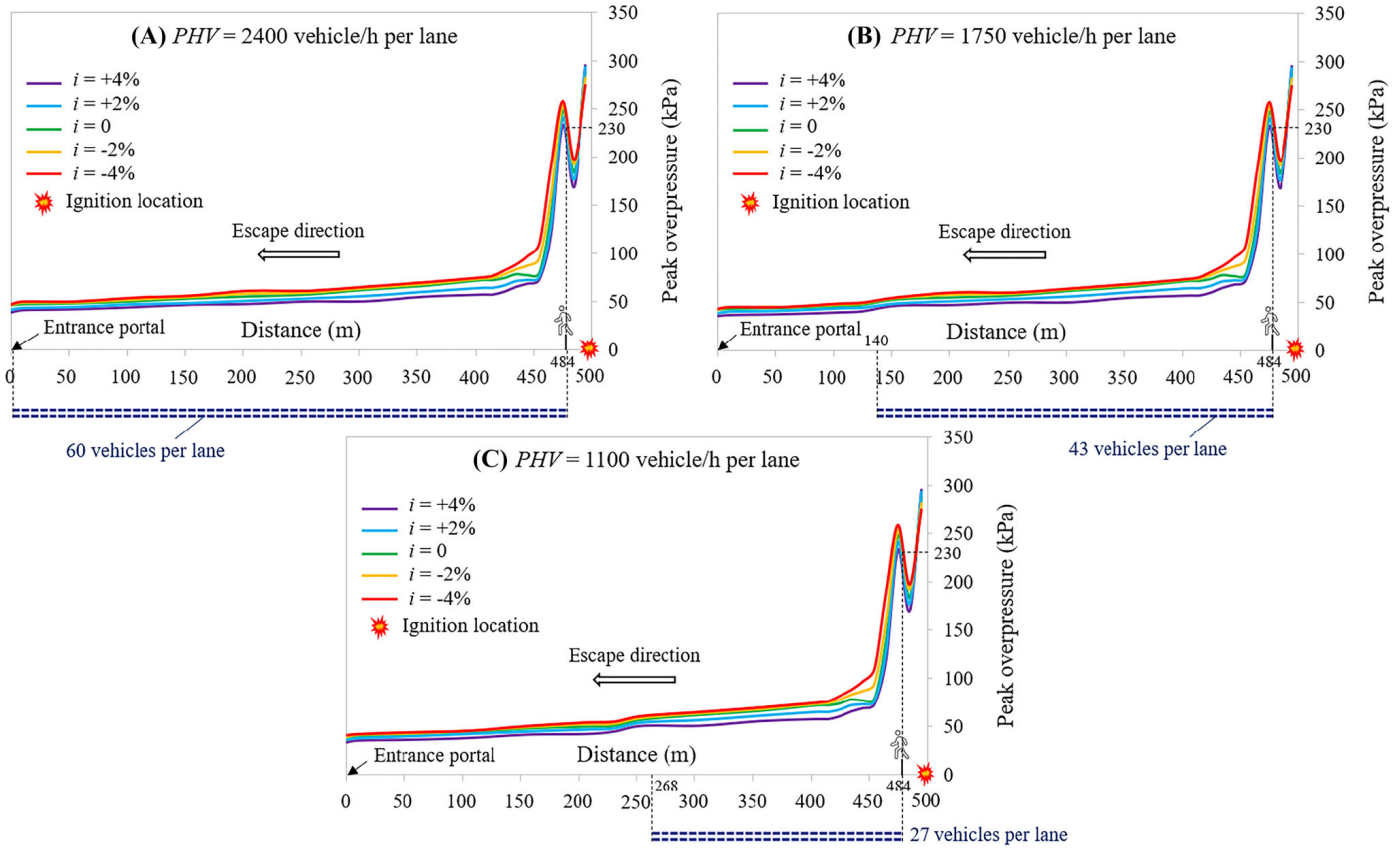
Peak overpressure decay upstream of the ignition location at the breathing height along the escape route of the 1 km long mechanically ventilated tunnel tube as a function of the longitudinal slope for different peak hourly volumes: (a) PHV = 2400 vehicles/h per lane; (b) PHV = 1750 vehicles/h per lane; (c) PHV = 1100 vehicles/h per lane.

By considering the 1 km long mechanically ventilated tunnel tube, Figure 18a−c shows the peak overpressure decay upstream of the ignition location along the escape route at the breathing height as a function of the longitudinal slope (i.e., *i* = +4, +2, 0, −2, or −4%) for a PHV of 2400, 1750, and 1100 vehicles/h per lane, respectively.

Like the case where only natural ventilation is present, we can see that the highest overpressures are found near the ignition location, with their gradual attenuation towards the entrance portal of the tunnel tube. However, it is to be stressed that the activation of the jet fans—compared to the case of natural ventilation—pushing the flammable hydrogen cloud more towards the exit portal of the tunnel tube, reduces the longitudinal extension of the mentioned zone near the ignition point where there are the highest overpressures, as well as it decreases the maximum value of the overpressure; finding, for example, that the user closest to the ignition location is subjected to a maximum overpressure of 230 kPa versus 265 kPa of the naturally ventilated tunnel tube.

Therefore, since it reduces the values of overpressure to which escaping people are exposed along their evacuation route, fewer potential victims are expected in the presence of mechanical ventilation.

#### 5.2.3 Peak Overpressure Decay Along the 2 Km Long Tunnel Tube Under Mechanical Ventilation

**FIGURE 19:**
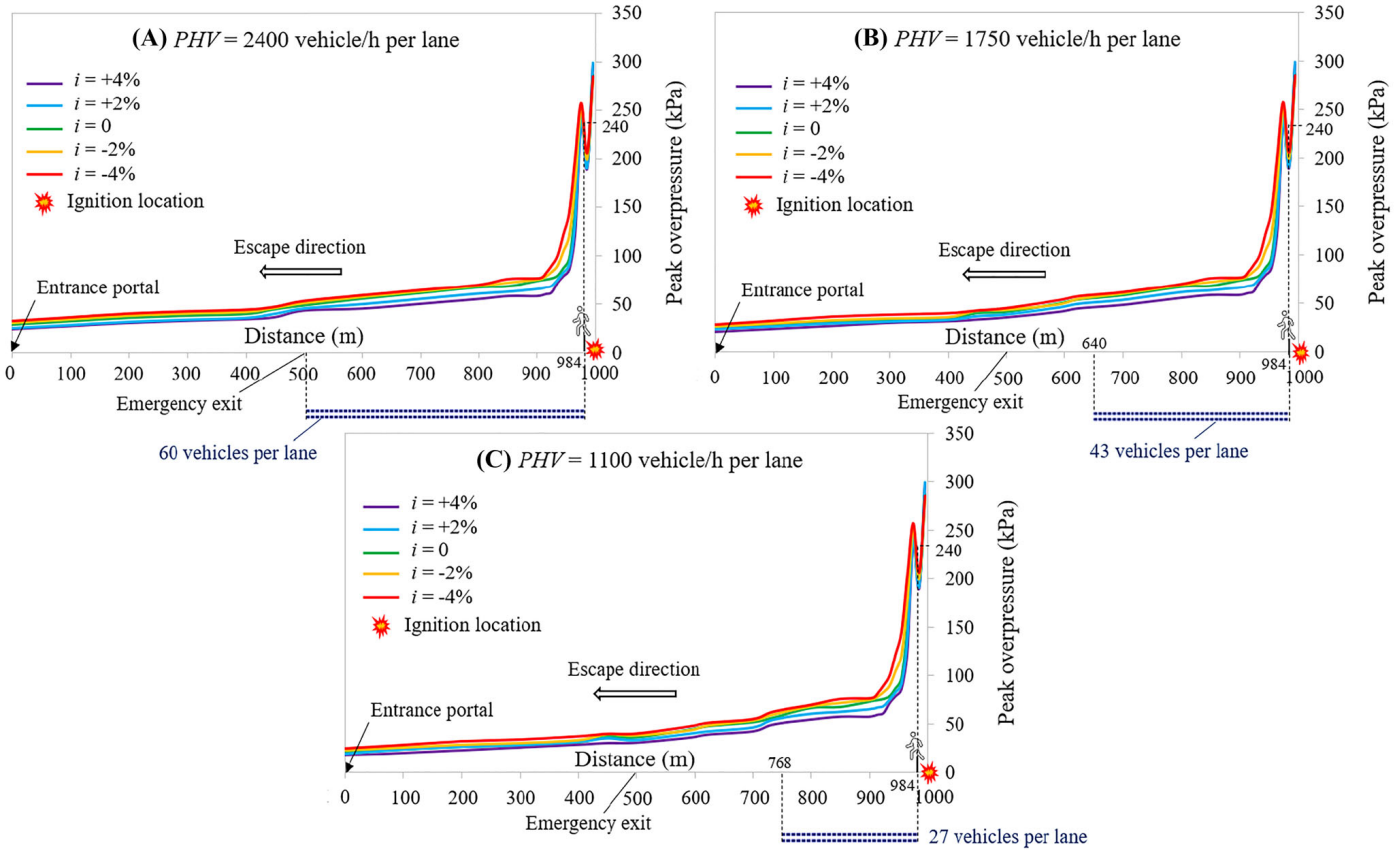
Peak overpressure decay upstream of the ignition location at the breathing height along the escape route of the 2 km long mechanically ventilated tunnel tube as a function of the longitudinal slope for different peak hourly volumes: (a) PHV = 2400 vehicles/h per lane; (b) PHV = 1750 vehicles/h per lane; (c) PHV = 1100 vehicles/h per lane.

With reference to the 2 km long mechanically ventilated tunnel tube, Figure 19a−c shows the peak overpressure decay upstream of the ignition location along the escape route at the breathing height as a function of the longitudinal slope (i.e., *i* = +4, +2, 0, −2, or −4%) for a PHV of 2400, 1750, and 1100 vehicles/h per lane, respectively. In this case, for a PHV equal to 2400 vehicles/h per lane only about 50% of the tunnel length upstream of the ignition location is filled by vehicles after 90 s from the release (i.e., the ignition time), while for a PHV of 1750 and 1100 vehicles/h per lane only about 35 and 22% of the tunnel length upstream the ignition site is occupied by vehicles, respectively.

Figure 19a−c also shows that the user closest to the ignition location is subjected to a peak overpressure slightly greater than that found for the 1 km long mechanically ventilated tunnel tube (i.e., 240vs. 230 kPa). Moreover, the region characterized by the greatest values of overpressure is found to extend a bit further upstream of the ignition location. This may be due to the fact that the steady‐state condition of the velocity of air flow in the longer tunnel, after the activation of the jet fans that push the flammable hydrogen cloud towards the exit portal, might be reached later compared to the 1 km long tunnel.

Therefore, it appears that the tunnel length could worsen user safety in the event of ignition of the LH_2_ released.

### 5.3 Safety Analysis

#### 5.3.1 Probit Function

There are various approaches that can be applied to evaluate the impact on user safety in the event of hazardous situations such as those involving hydrogen–air mixture explosions. Among these, probit functions are widely used in risk assessment, providing the probability of injury or death as a function of the severity of an accident scenario. Given that the main effects on people in the case of an explosion are associated with the consequences generated by overpressures, the probit function developed by the Health and Safety Executive (HSE) (LaChance et al. 2011; Health and Safety Executive 2013) was selected in this study to assess the safety of tunnel occupants in case of ignition of the flammable hydrogen cloud. The analytical equation is as follows:

(1) $$Y=5.13+1.37\ln\left(P\right)$$

where *Y* is the probit function and *P* is the peak overpressure in bar.

The probability of fatality ($P_{\mathrm{fatality}}$) due to lung hemorrhage of each individual is then related to the probit function (Y) as follows:

(2) $$p_{\mathrm{fatality}}=50\left[1+\frac{Y-5}{\left|Y-5\right|}\mathrm{ERF}\left(\frac{\left|Y-5\right|}{\sqrt{2}}\right)\right]$$

where ERF stands for the Gaussian error function.

#### 5.3.2 Number of Potential Fatalities

For each scenario investigated, the number of potential fatalities caused by the hydrogen–air mixture explosion was computed by combining the previous results in terms of overpressures with the probit function developed by the Health and Safety Executive (2013). For this aim, the first step consisted of determining the exact position occupied by each escaping user at the ignition time of the flammable hydrogen cloud, then the number of individuals exposed to a given overpressure and the associated probability of death were calculated (see the Appendix).

#### 5.3.3 Probability Matrix of the Risk of Having a Given Number (*N*) of Fatalities

The probability matrix of the risk of having a given number of potential fatalities (*N*), which is reported in Table 4, show the results obtained as a function of the tunnel length (*L*), longitudinal slope (*i*), PHV, and type of ventilation (i.e., natural or longitudinal mechanical). From Table 4, it is possible to observe that the probability of having a given number of potential fatalities within the tunnels investigated significantly increases as the longitudinal slope becomes negative (i.e., passing from +4% to −4%). For example, with reference to the 1 km long naturally ventilated tunnel under congested traffic conditions (i.e., PHV = 2400 vehicles/h per lane), the probability of having 10, 100, or 200 potential fatalities was found to increase from 81 to 93%, from 26 to 43%, or from 20 to 33% passing from *i* = +4 to *i* = −4%. However, if the 1 km long tunnel tube is equipped with a longitudinal mechanical ventilation system, the probabilities of having 10, 100, or 200 potential fatalities under congested traffic conditions decrease, confirming the benefits of ventilation. These findings are consistent with previous studies of the literature (e.g., Bie and Hao 2017; Hu et al. 2023; Cui et al. 2023; Yan et al. 2024), which have demonstrated that ventilation significantly influences hydrogen dispersion and limits the formation of large combustible clouds, thereby reducing peak overpressures in the event of an explosion. It should be stressed that while the aforementioned papers did not investigate the effects of tunnel geometry and traffic, our study shows that the growth trend in the probability of having a given number of potential fatalities is non‐linear by reducing the positive longitudinal slope up to the negative one, as well as by varying the longitudinal slope the reduction in the probability of fatalities is also non‐linear by passing from the naturally to the mechanically ventilated tunnel.

**TABLE 4:** Probability matrix of the risk of having a given number of fatalities *N* as a function of the tunnel length (*L*), longitudinal slope (*i*), Peak Hourly Volume (PHV), and type of ventilation (i.e., natural or longitudinal mechanical).

		Probability of potential fatalities *N* (*p* [%])																				
		*L* = 1 km														*L* = 2 km						
Type of ventilation		Natural							Longitudinal mechanical							Longitudinal mechanical						
**Potential fatalities *N***		1	10	50	100	150	200	240	1	10	50	100	150	200	240	1	10	50	100	150	200	240
**PHV = 2400 vehicles/h per lane**	**i = −4%**	94	93	53	43	36	33	30	93	86	37	31	25	21	18	93	90	43	35	30	26	23
	**i = −2%**	93	91	48	39	33	29	27	92	83	35	29	24	20	17	93	86	40	33	28	24	21
	**i = 0**	93	90	42	34	29	25	24	92	80	32	26	22	18	16	92	83	37	31	25	21	19
	**i = +2%**	92	86	38	30	25	22	21	91	70	29	23	20	16	14	92	75	34	26	22	19	16
	**i = +4%**	91	81	33	26	22	20	19	90	57	25	21	18	14	12	91	61	30	24	20	18	15
**PHV = 1750 vehicles/h per lane**	**i = −4%**	94	93	53	43	36	—	—	93	86	37	31	25	—	—	93	90	43	36	30	—	—
	**i = −2%**	93	91	48	39	33	—	—	92	83	35	29	24	—	—	93	86	40	33	28	—	—
	**i = 0**	93	90	42	34	29	—	—	92	80	32	26	22	—	—	92	83	37	31	25	—	—
	**i = +2%**	92	86	38	30	25	—	—	91	70	29	23	20	—	—	92	75	34	26	22	—	—
	**i = +4%**	91	81	33	26	22	—	—	90	57	25	21	18	—	—	91	61	30	24	20	—	—
**PHV = 1100 vehicles/h per lane**	**i = −4%**	94	93	53	43	—	—	—	93	86	37	31	—	—	—	93	90	43	35	—	—	—
	**i = −2%**	93	91	48	39	—	—	—	92	83	35	29	—	—	—	93	86	40	33	—	—	—
	**i = 0**	93	90	42	34	—	—	—	92	80	32	26	—	—	—	92	83	37	31	—	—	—
	**i = +2%**	92	86	38	30	—	—	—	91	70	29	23	—	—	—	92	75	34	26	—	—	—
	**i = +4%**	91	81	33	26	—	—	—	90	57	25	21	—	—	—	91	61	30	24	—	—	—

Moreover, by comparing the 2 km long mechanically ventilated tunnel tube with the corresponding 1 km tunnel tube, it is to be noted that under the same longitudinal slope and traffic volume, the probability of having a certain number of potential fatalities increases.

Therefore, the findings showed that the risk—in terms of the probability (*p*) of having a given number of potential fatalities *N*—decreases in the presence of mechanical ventilation and lower traffic volumes, while increases with the negative longitudinal slope and tunnel length.

With reference to the negative longitudinal slope, which has a greater impact on the increase of the probability (*p*) of having a given number of potential fatalities (*N*) compared to the tunnel length and traffic volume, this is due to the so‐called inverse chimney effect, which can highly worsen conditions for tunnel users upstream of the LH_2_ release point in the tunnel.

Under the same mechanical ventilation, slope and traffic volume conditions, the probability (*p*) of having a given number of potential fatalities (*N*) increases with the length of tunnel due to the reduced availability of open spaces for the LH_2_ to dissipate into the atmosphere.

Finally, with lower traffic volumes that act as obstacles to the spread of the LH_2_ along the tunnel, the aforementioned probability (*p*) is also expected to reduce.

## 6 Conclusions

This study aimed to investigate the combined effect of the geometric, traffic, and ventilation characteristics of a unidirectional road tunnel on user safety in the event of accidental releases and possible explosions of LH_2_ from a road tanker. For this purpose, specific 3D CFD models of LH_2_ releases and explosions were developed using the ANSYS Fluent code, and a comprehensive parametric analysis was carried out by varying the tunnel length (1 and 2 km), the longitudinal slope (+4, +2, 0, −2, and −4%), the traffic volume (1100, 1750, and 2400 vehicles/h per lane), and the type of ventilation (i.e., natural or longitudinal mechanical due to the presence of jet fans). To prove its ability to reliably replicate actual scenarios, the ANSYS Fluent code was preliminary calibrated against experimental tests reported in literature involving both LH_2_ releases and explosions.

Regarding the release simulations, the results showed that the spatial distance reached by the flammable cloud upstream of the spill site decreases with the longitudinal mechanical ventilation compared to the natural one, while increasing as the longitudinal slope (*i*) of the tunnel tube becomes negative (i.e., from +4 to −4%) due to the inverse chimney effect. Moreover, under longitudinal mechanical ventilation, as the tunnel length increases, a slightly greater propagation of the flammable hydrogen cloud upstream of the release is found.

By considering the most critical condition for the ignition time of the hydrogen‐air mixture (i.e., after 90 s from the release of LH_2_), the explosion simulations showed that: (i) higher overpressures are found in close proximity to the ignition point, then gradually decrease towards the entrance portal of the tunnel tube; (ii) under the same PHV, the region with the highest overpressure extend further upstream of the ignition location as the longitudinal slope of the tunnel tube decreases (i.e., passing from +4 to −4%); (iii) for a given gradient, lower overpressures are simulated at the entrance portal of the tunnel tube as the PHV decreases due to the reduced vehicle's queue length that implicates a larger cross‐sectional area without obstacles when compared to congested traffic conditions, which leads to a greater dissipation; (iv) the average values of the overpressures upstream of the ignition site are lower in the presence of a longitudinal mechanical ventilation compared to the naturally ventilated tunnel, which is due to the activation of the jet fans that push the flammable hydrogen cloud more towards the exit portal; (v) under the longitudinal mechanical ventilation conditions, as the tunnel length increases, the tunnel region characterized by the highest overpressures was found to extend a bit further upstream of the ignition site.

The probability matrix of the risk summarizes the results obtained: the probability of having a given number of potential fatalities within the tunnels investigated significantly increases with negative gradients, while decreasing in the presence of jet fans, thus confirming the benefits of implementing a longitudinal mechanical ventilation system. In this regard, even if a mechanical ventilation system may be supposed to be activated only with the occurrence of the LH_2_ release, the economic feasibility and real‐world implementation aspects, including the cost‐benefit analysis associated with the mechanical ventilation in road tunnels should also be addressed. Finally, the probability of having a certain number of potential fatalities increases with the tunnel length.

By providing additional knowledge in the field of hydrogen safety, this study might serve as a reference for tunnel operators in the choice of mitigation measures and/or traffic control strategies to limit the negative consequences of the release of LH_2_ in road tunnels.

Although this study has increased the knowledge on the safety of hydrogen transportation through tunnels, there are still some points that are worth investigating. While this study focused on the effects of mechanical ventilation on the tunnel safety in the event of LH_2_ release, when compared to the natural one, as a function of geometry and traffic conditions; and in addition by developing a probabilistic risk matrix it provides practical applications for tunnel operators, future works should also explore the influence of human behavior, including evacuation response and decision‐making during LH_2_ release events. Therefore, further studies are necessary for making additional developments in the field of research on this topic. A future extension of research might also be to investigate many more tunnels characterized by a multitude of different geometries and traffic levels for building a significant sample of data to implement in a statistical analysis for capturing certain possible nonlinear interactions among the variables investigated. As a result, additional studies on the topic investigated are necessary.

## Conflicts of Interest

The authors declare no conflicts of interest.

## Figures and Tables

**FIGURE A1:**
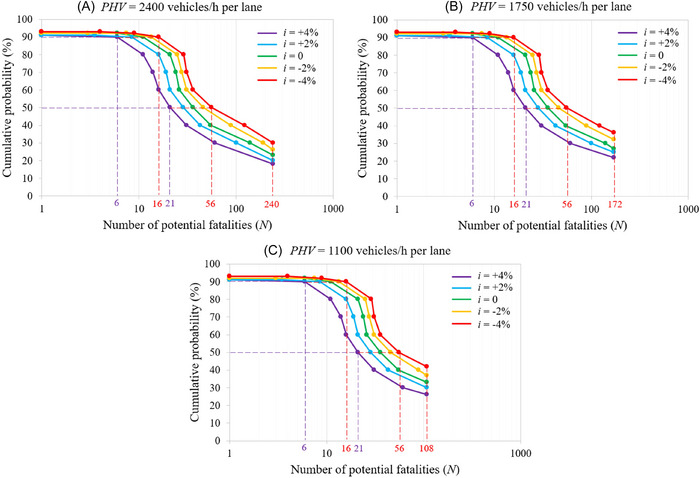
Cumulative probability of *N* or more potential fatalities within the 1 km long naturally ventilated tunnel tube as a function of the longitudinal slope for different peak hourly volumes: (a) PHV = 2400 vehicles/h per lane; (b) PHV = 1750 vehicles/h per lane; (c) PHV = 1100 vehicles/h per lane.

**FIGURE A2:**
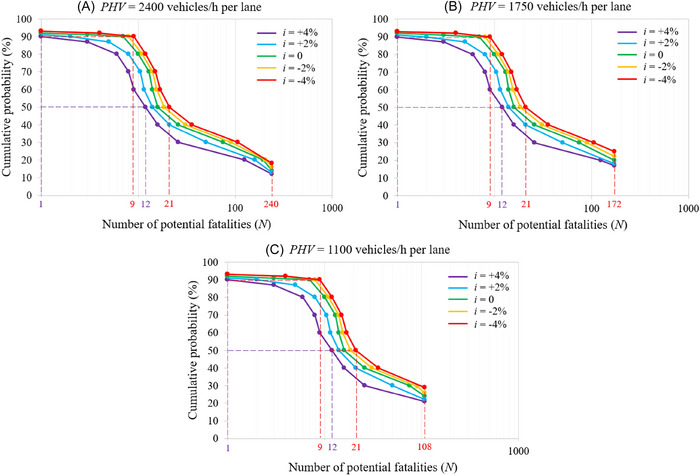
Cumulative probability of *N* or more potential fatalities within the 1 km long mechanically ventilated tunnel tube as a function of the longitudinal slope for different peak hourly volumes: (a) PHV = 2400 vehicles/h per lane; (b) PHV = 1750 vehicles/h per lane; (c) PHV = 1100 vehicles/h per lane.

**FIGURE A3:**
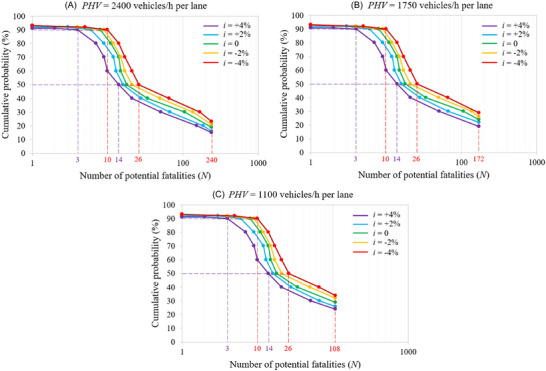
Cumulative probability of *N* or more potential fatalities within the 2 km long mechanically ventilated tunnel tube as a function of the longitudinal slope for different peak hourly volumes: (a) PHV = 2400 vehicles/h per lane; (b) PHV = 1750 vehicles/h per lane; (c) PHV = 1100 vehicles/h per lane.

## Appendix 1

### 1.1

Figure A1, Figure A2, and Figure A3 show the cumulative probability of *N* or more fatalities as a function of both the PHV and longitudinal slope within the 1 km long tunnel tube under natural ventilation, as well as the 1 and 2 km long tunnel tubes equipped with a mechanical ventilation system, respectively. In all the cases investigated, for a fixed probability of death, it was found that the number of potential fatalities significantly increases as the gradient becomes negative (i.e., passing from +4% to −4%). For example, regarding the 1 km long naturally ventilated tunnel tube with a PHV of 2400 vehicles/h per lane (Figure A1a), the number of tunnel users with a 90% probability of death increases from 6 to 16 passing from *i* = +4% to *i* = −4%, while those with 50% probability of fatality rise from 21 to 56. This result is consistent with those in terms of overpressures, the average value of which along the escape route was always found to increase as the tunnel inclination decreased from +4% to −4%.

By comparing Figure A1 and Figure A2, it can also be noted that the number of potential fatalities is significantly lower when the 1 km long tunnel is assumed to be mechanically instead of naturally ventilated, considering all the PHV*s* and gradients under investigation. As an example, for PHV = 2400 vehicles/h per lane and *i* = −4%, the number of people having a 90% and 50% probability of fatality reduces from 16 to 9 and from 56 to 21 by equipping the 1 km long tunnel with a longitudinal mechanical ventilation system, respectively. This result is also consistent with those in terms of overpressures, the intensity of which along the escape route was always found to be mitigated by the activation of the jet fans.

Lastly, by comparing Figure A2 and Figure A3, under mechanical ventilation conditions, the number of potential fatalities also increases as the tunnel length increases, considering all the PHV*s* and longitudinal slopes under investigation. For example, for PHV = 2400 vehicles/h per lane and *i* = −4%, the number of people with a 90% and a 50% probability of death increases from 9 to 10 and from 21 to 26 when the tunnel length passes from 1 to 2 km, respectively. This result reflects those in terms of overpressures, indicating how the tunnel length might have a negative impact on user safety. Since the highest overpressures are found within a zone near the ignition location, the length of which—at the ignition time (90 s)—is less than the length of vehicles in the queue for all the PHVs considered, it is expected that the probability of having a given number of fatalities in that area is the same independent of the traffic levels investigated (see, for example, the 50% and/or 90% probability of occurrence). Nevertheless, the total number of occupants in the tunnel tube upstream of the ignition location is different at the ignition time due to the different number of queued vehicles for the three traffic levels considered; as a result the total number of potential fatalities, even if some of them are characterized by the lowest values of the probability of occurrence, decreases from 240 to 172 up to 108 for a PHV of 2400, 1750, and 1100 vehicles/h per lane, respectively.
